# MicroRNAs Dynamically Remodel Gastrointestinal Smooth Muscle Cells

**DOI:** 10.1371/journal.pone.0018628

**Published:** 2011-04-14

**Authors:** Chanjae Park, Wei Yan, Sean M. Ward, Sung Jin Hwang, Qiuxia Wu, William J. Hatton, Jong Kun Park, Kenton M. Sanders, Seungil Ro

**Affiliations:** 1 Department of Physiology and Cell Biology, University of Nevada School of Medicine, Reno, Nevada, United States of America; 2 Division of Biological Science, Wonkwang University, Iksan, Chonbuk, South Korea; Virginia Commonwealth University, United States of America

## Abstract

Smooth muscle cells (SMCs) express a unique set of microRNAs (miRNAs) which regulate and maintain the differentiation state of SMCs. The goal of this study was to investigate the role of miRNAs during the development of gastrointestinal (GI) SMCs in a transgenic animal model. We generated SMC-specific *Dicer* null animals that express the reporter, green fluorescence protein, in a SMC-specific manner. SMC-specific knockout of *Dicer* prevented SMC miRNA biogenesis, causing dramatic changes in phenotype, function, and global gene expression in SMCs: the mutant mice developed severe dilation of the intestinal tract associated with the thinning and destruction of the smooth muscle (SM) layers; contractile motility in the mutant intestine was dramatically decreased; and SM contractile genes and transcriptional regulators were extensively down-regulated in the mutant SMCs. Profiling and bioinformatic analyses showed that SMC phenotype is regulated by a complex network of positive and negative feedback by SMC miRNAs, serum response factor (SRF), and other transcriptional factors. Taken together, our data suggest that SMC miRNAs are required for the development and survival of SMCs in the GI tract.

## Introduction

Dicer is a key endonuclease in the RNA interference machinery that cleaves precursor microRNAs (miRNAs) into mature miRNAs during miRNA biogenesis [1], [2]. MicroRNAs are post-transcriptional regulators that play critical roles in animal development. Tissue or cell-specific knockout studies of *Dicer* have been performed to uncover the functions of miRNAs during the development of cells including embryonic stem cells [3], female germline cells [4], testis Sertoli cells [5], pancreatic islet cells [6], lung epithelial cells [7], and skeletal chondrocytes [8]. These studies show that miRNAs are essential in cell proliferation and/or differentiation during development.

Smooth muscle cells (SMCs), which provide motor activity in the blood vessels, airways, the bladder, urinary tract, and gastrointestinal (GI) tract, exhibit remarkable phenotypic plasticity in congenital or acquired pathological conditions [9], [10]. Phenotypic changes in SMCs result from substantial changes in gene expression driven largely by serum response factor (SRF) [10]–[14]. SRF acts as a master switch for the expression of contractile and cytoskeletal genes in virtually all cells across diverse species [15]. The activity of SRF is regulated through interactions with transcriptional co-activators such as myocardin, the myocardin family members MRTFA and MRTFB [16], [17], as well as co-repressors such as ELK1 [18], NF-kB [19], and HERP1 [20]. During SMC differentiation, SRF regulation is achieved by a CArG [CC (A/T)_6_ GG] box found principally in the promoter and intronic regions of virtually all SM-restricted genes [12], [13]. SRF binds to the CArG box, which then transcriptionally activates or represses SM genes, depending on its associated cofactors.

Recent studies have shown that miRNAs are able to regulate SMC proliferation and/or differentiation. A set of miRNAs including miR-21, miR-133a, miR-143, and miR-145 have been identified as regulators of SMC growth and/or differentiation in cardiovascular disorders [21]–[25]. Interestingly, some of these SM miRNAs are known to be regulated by SRF through conserved CArG boxes [22], [24], [25].

We have previously identified GI SM miRNA transcriptomes from mice and humans which are highly conserved in both organisms [26]. The SM miRNA transcriptomes consist of several hundreds of unique miRNAs which comprise almost half of the entire 701 miRNAs in the miRBase registry [27]. We found that SMC differentiation and proliferation are modulated by SRF-dependent, SMC-phenotypic miRNAs. These findings suggest that SMC miRNAs play an important role during the development of SMCs in the GI tract. Vascular smooth muscle (VSM)-specific *Dicer*-null mice have shown dilated, thin-walled blood vessels that were affected by a reduction in SMC proliferation [28].

Here we show how the loss of miRNAs affects GI SMC phenotype in the SMC-specific *Dicer* null animal. These mice display severe morphological, functional, and genetic defects in GI SMCs, which as a whole present the features of the chronic intestinal pseudo-obstruction (CIPO) or the congenital human megacystis-microcolon-Intestinal hypoperistalsis syndrome (MMIHS). Both CIPO and MMIHS are characterized by a dilated intestine with air-fluid levels as well as impaired intestinal motility in the absence of a mechanical obstruction [29], [30]. Here we propose a model for the regulation of SMC phenotype by a complex network of indirect and direct positive and negative feedback loops by SM miRNAs, SRF, and additional transcriptional factors. This model provides new insight into the interaction between miRNAs and their target genes in SMCs during development, and therefore provides a new basis for understanding the development of GI disorders based on muscle myopathy.

## Materials and Methods

For details, see **Text S1** for extended Materials and Methods.

### Generation of *smDicer* mutant mice

SMC-specific *Dicer* null *smDicer^−/−;Cre-GFP/+^* (*smDicer* mutant) mice were generated by cross-breeding a *smMHC/Cre/eGFP* (*smMHC^Cre-GFP/+^*) male mouse (gift from Kotlikoff M.I. at Cornell University) [31] and a *Dicer^lox/lox^* female homozygote mouse (The Jackson Laboratory) according to a procedure approved by the Institutional Animal Care and Use Committee at the University of Nevada, Reno.

### Tissue preparation

The *smDicer* mutant, wild type (WT) *smMHC^Cre-GFP/+^*, or C57BL6 mice were anesthetized by isoflurane inhalation and euthanized by decapitation at the age of ∼3 weeks. Multiple tissues were dissected from the mice. The GI tissues were stripped free of mucosa and submucous plexus. The multiple tissues were used for the isolation of total RNAs. Some of the small intestinal and colonic muscularis tissues were used for Western blots or purification of SMCs using flow cytometry and fluorescence-activated cell sorter (FACS). Small intestinal muscularis from ∼3-weeks-old C57BL6 mice were used for organ culture. The GI tissues were used for microscopic histological analysis or for mechanical response studies.

### Isolation of total and small RNAs from tissues and cells

Total RNAs and/or small RNAs were isolated from multiple tissues and sorted SMCs from the *smDicer* mutant and the WT control mice using the mirVanaTM miRNA isolation kit (Ambion) as described [32].

### RT-PCR and qPCR detection of messenger and small RNAs

Preparation of the cDNA libraries from the total RNAs isolated from multiple tissues and from sorted SMCs obtained by FACS, as well as qPCR analysis on cDNAs, were performed as described [33]. Preparation of the small RNA cDNA (srcDNA) libraries obtained from the small RNAs isolated from small intestine tissues and from SMCs sorted by FACS, as well as qPCR analysis on srcDNAs, were performed as described [34]. All primers used for RT-PCR and qPCR are shown in Table S1.

### Western blot analysis

SRF and ACTA2 (SM α-actin) expression in the small intestinal and colonic tissues from the *smDicer* mutant and the WT control mice were analyzed by Western blot as described [26]. The expression level of ACTA2 and SRF was normalized by GAPDH.

### Confocal microscopy and histological analysis

Enhanced green fluorescent protein (eGFP) fluorescence microscopic analysis of the small intestine, cecum, and colon was performed as described [35]. For histological analysis, colonic tissues from the *smDicer* mutant and the WT control mice were dehydrated, embedded in paraffin, cut into 6 µm-thick coronal sections, rehydrated, and stained with hematoxylin and eosin (H & E). Images were collected using the Zeiss confocal microscope.

### Flow cytometry and fluorescence-activated cell sorting

Single cell suspensions from small intestinal and colonic muscularis dissected from the *smDicer* mutant and the WT control mice were prepared as described [35]. Cells were analyzed and sorted (130 µm nozzle, 12 psi) on a Becton Dickinson FACSAria II (Becton Dickinson). FlowJo software (Tree Star) was used to analyze the data. Sorted cells were used for the isolation of total and small RNAs.

### GeneChip arrays for messenger RNAs

Mouse mRNA microarrays were performed on GeneChip® Mouse Genome 430 2.0 Arrays (Affymetrix) covering 39,000 mouse gene transcripts and variants. About 2 µg of total RNAs isolated from the small intestinal smooth muscles of the *smDicer* mutant and the WT control mice (2 each) were used for the arrays. The cDNA synthesis, hybridization, cDNA labeling, and scanning were performed at the Nevada Genomic Center (Reno). Array data were analyzed by Phalanx Biotech Group (Palo Alto) using Omicsoft Array Studio software and normalized using the RMA algorithm. Analyzed array datasets were deposited at the NCBI Gene Expression Omnibus (GEO) (GSE21738).

### Analysis of mechanical response

Small intestine and proximal coloic tissues (10 mm) from the *smDicer* mutant and WT control mice were fixed at one end and attached to a Fort 10 isometric force transducer (WPI). Isometric force was measured as described [36]. All experiments were performed in the presence of L-NNA (100 µM) to eliminate the possible contribution from nitrergic nerve activity.

### Analysis of targeting microRNAs

Potential targeting miRNAs for 97 dysregulated SM genes and 58 dysregulated transcription regulators were retrieved and analyzed from two well-established target prediction programs: PicTar [37] and TargetScan (release 5.1) [38], [39]. Genechip array data (fold change and P value) and expression profiles (SM-common, SM-unique, and non-SM miRNAs) were shown along with PicTar and TargetScan miRNAs. Total hit miRNAs and average miRNAs per gene in SM-common, SM-unique, and non-SM miRNAs were calculated from PicTar and TargetScan miRNAs.

## Results

### Phenotypic changes in the SMC-specific *Dicer* null mice

**Figure 1:**
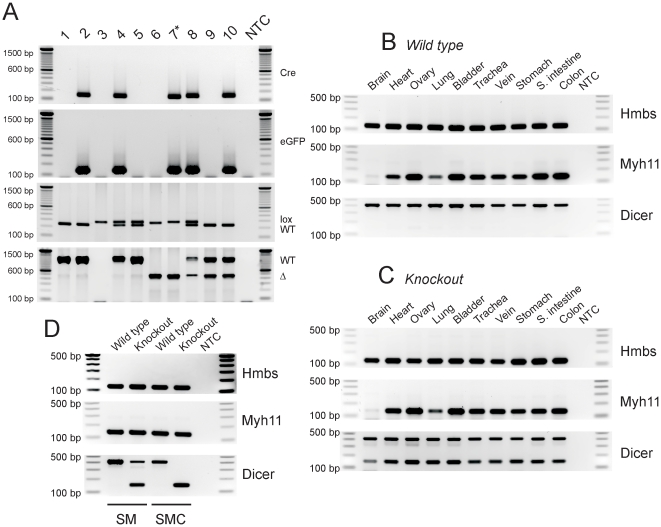
Genotyping and expression analysis of SMC-specific *Dicer* knockout mice. (**A**) Each mouse line was genotyped using PCR with a set of primers specific for *Cre*, *eGFP*, inserted lox P sites (lox), and wild type (WT) or deleted locus (Δ). The *smDicer* homozygous knockout (KO) *smDicer^−/−;Cre-GFP/+^* is indicated by an asterisk on the mouse line 7 (★). All genotypes for mouse lines 1–10 are shown in Table 1. (**B**) *Myh11* and *Dicer* transcripts abundantly expressed in the SM organ tissues from the WT mouse. *Hmbs* was used as an endogenous control. (**C**) Expression of *Myh11* and *Myh11*-dependent KO (deletion) of *Dicer* transcripts in the SM organ tissues from the *smDicer* KO mouse. Note that the deleted *Dicer* PCR products (∼150 bp) are smaller than the WT (∼400 bp). (**D**) Expression of *Myh11* and *Dicer* transcripts in the small intestine SM tissue (SM) and cells (SMCs) sorted by FACS. *Dicer* transcripts were completely deleted in the sorted SMCs from the *smDicer* KO mouse while they were partially deleted in the SM. All PCR products were analyzed on 1.5% agarose gels along with a DNA ladder. NTC stands for non-template control, SM for smooth muscle tissue, and SMC for smooth muscle cells.

**Figure 2:**
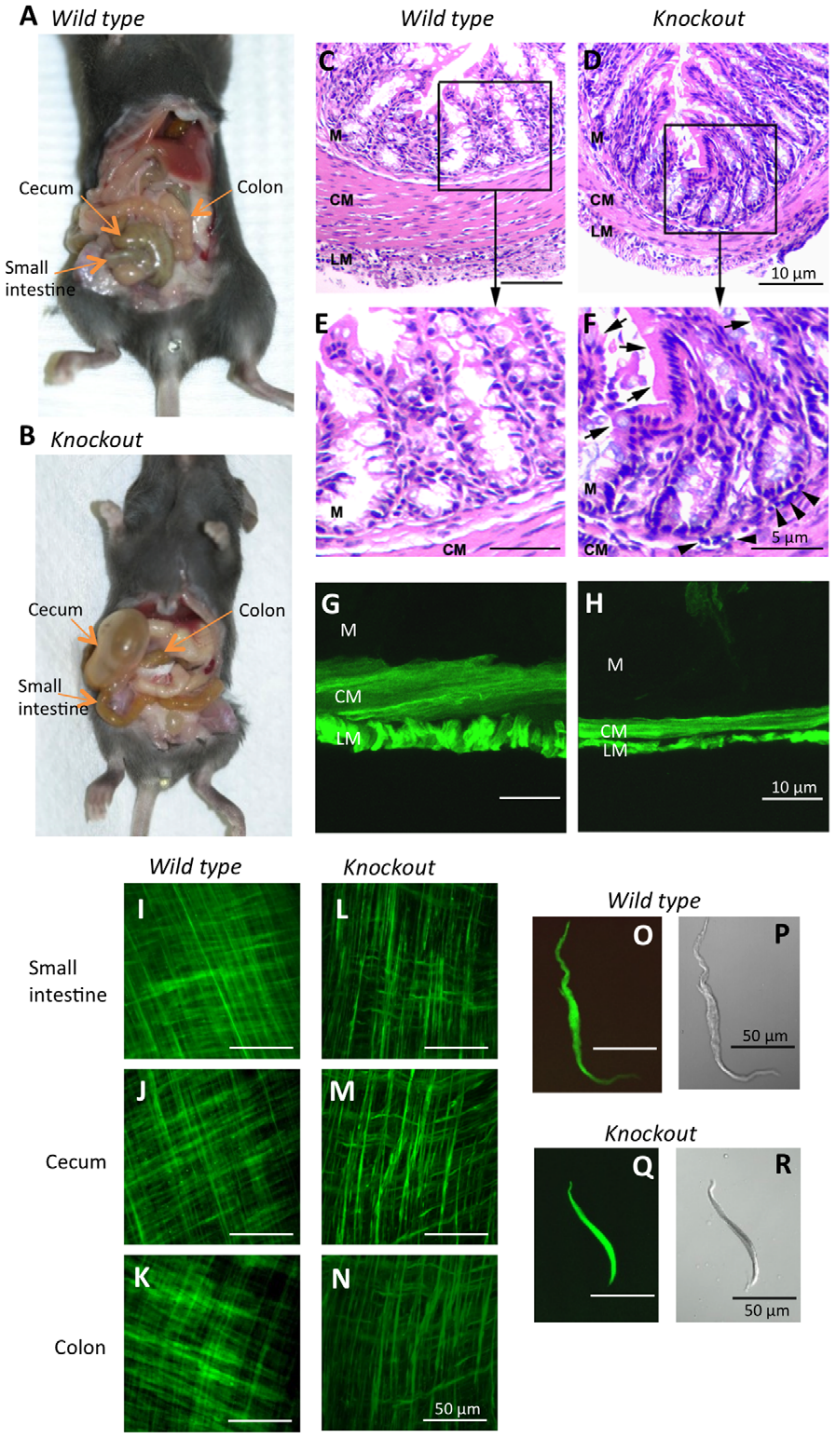
Phenotypic changes of SMCs in the *smDicer* knockout mice. (**A, B**) The gross morphological changes in a 20 day old *smMHC^Cre-GFP/+^* (wild type) mouse (A) compared to a *smDicer^−/−;Cre-GFP/+^* (*smDicer* knockout) mouse (B) of the same age. Note the large, distended cecum as well as the partially distended antrum, small intestine, and colon in the *smDicer* knockout mutant. (**C, D**) Representative digital photomicrographs of cryostat sections from the colon of wild type control and mutant mice respectively (hematoxylin and eosin staining). (C) A wild type mouse shows unremarkable histology through the entire thickness of the colonic wall. (D) A *smDicer* knockout mouse shows that both the circular (CM) and longitudinal (LM) muscle layers of the colonic muscularis appear atrophied compared to the wild type mouse. Also noticeable is that the mucosal (M) villi in the knockout mouse appear blunted, thickened, inflamed, and accompanied by a chronic inflammatory infiltrate. (**E, F**) Enlarged regions of C and D showing that in the *smDicer* knockout section (**F**) villi are blunted, thickened, and inflamed (arrows), and that there are numerous cells (arrowheads) constituting an inflammatory infiltrate indicative of chronic inflammation. (**G, H**) LSCM and DIC images of cryostat sections taken from the small intestine of the control (G) and *smDicer* knockout (H) under UV. Note that both the CM and LM are much thinner in the knockout compared to the controls. (**I–N**) LCSM images of the entire thickness of the muscularis of the control small intestine (I), cecum (J), and colon tissue (K) from the knockout mouse. LCSM images of the entire thickness of the muscularis from the small intestine (L), cecum (M), and colon tissue (N) of the knockout showing less well-differentiated SMC organization. (O–R) Fluorescence and contrast phase images of an isolated intestinal SMC from the control (O and P) and knockout (Q and R). Note the disorganized and abnormal appearance of the circular and longitudinal SMCs from the knockouts compared to the controls.

A previous study showed that loss of *Dicer* in vascular SMC resulted in embryonic arrest and defective SMC contractile and cytoskeletal organization [28]. We generated SMC-specific *Dicer* null (*smDicer^−/−;Cre-GFP/+^*)mice by cross breeding *smMHC/Cre/eGFP* (*smMHC^Cre-GFP/+^*) [31] and *Dicer^lox/lox^*, in which mature miRNAs were depleted in all SMCs. The *smMHC^Cre-GFP/+^* mice express *Cre* recombinase and *eGFP*, which are directed by the SM myosin heavy chain (*Myh11*) promoter and show SMC-specific *Cre/eGFP* expression in SM organs [31]. All mice generated for this study are summarized in Table 1. Each mouse was genotyped with a set of primers (see Table S1) specific for Cre, eGFP, inserted lox P sites (lox), and for wild type (WT) or deleted locus (Δ) (Figure 1A). *Myh11* and *Dicer* transcripts were abundantly expressed in the nine SM organ tissues from the WT mouse (Figure 1B). *Myh11*-dependent knockout (KO, deletion) of *Dicer* transcripts in the SM organ tissues from the *smDicer^−/−;Cre-GFP/+^* (*smDicer* mutant) mice resulted in partially deleted *Dicer* PCR products (∼150 bp) which were smaller than the WT (∼400 bp) (Figure 1C). We previously demonstrated small intestinal and colonic SMCs sorted by FACS from *smMHC^Cre-GFP/+^* mice were a pure population of differentiated SMCs [26]. The same FACS technique was used to sort mutant (KO) SMCs from the *smDicer* mutant mice along with WT SMCs from the WT controls. Sorted KO SMCs were lacking WT *Dicer* PCR products which were detected in WT SMCs, whereas they were partially deleted in SM tissues where other cells would still express *Dicer* (Figure 1D), confirming *Dicer* was completely deleted in KO SMCs. *smDicer* WT and heterozygous mice showed no visible phenotypic changes (Table 1). However, *smDicer* homozygous KO mice showed an extreme GI phenotype and died at ∼3 weeks of age. The *smDicer* mutant mice were smaller and weighed 73% of its WT or heterozygote siblings measured at 20–21 days (n = 5). The average length of the *smDicer* mutant small intestine (15.0 cm) and colon (2.9 cm) was significantly shorter than the small intestines (18.2 cm) and colons (4.0 cm) from the WT or heterozygote siblings. However, the normalized length of the small intestine (2.2 cm/g for KO and 2.0 cm/g for WT) and colon (0.4 cm/g for KO and 0.4 cm/g for WT) by weight between the *smDicer* KO and WT was similar, indicating the shorter small intestine and colon of the *smDicer* KO was due to less body weight. The cecum was significantly distended in the mutant while the antrum, small intestine, and colon appeared to be partially distended (Figure 2B) compared to those of the WT control mouse (Figure 2A). The circular and longitudinal muscle layers of the *smDicer* mutant colon (Figure 2D) were significantly atrophied, measuring only 44% of the WT colon (Figure 2C). Inflammation of the villous lining within the mucosal layer was also observed, characterized by blunting and thickening of the villi (Figure 2F) when compared to WT (Figure 2E). GFP expression and thickness of the muscularis of the small intestine in the *smDicer* mutant mouse (Figure 2H) when compared to WT (Figure 2G) were further confirmed using both laser scanning confocal microscopy (LSCM) and differential interference contrast (DIC) microscopy. The SM bundles of both layers from the GI tracts (small intestine, cecum, and colon) of the *smDicer* mutant were noticeably disorganized and underdeveloped (Figure 2L–N) compared to those of the control mouse (Figure 2I–K). The single SMC of the *smDicer* mutant appeared smaller and abnormal (Figure 2Q and R) compared to the control (Figure 2O and P). The morphological data obtained from SMCs would suggest that there is a reduction in the number and size of cells, which would lead to a thinner *tunica muscularis* in the *smDicer* mutant mice.

**Table 1:** Summary of double transgenic mice generated in this study.

	Genotype	Total	Male	Female	smDicer	Phenotype
1	*Dicer^+/+^;smMHC^+/+^*	7	3	4	Wild type	Normal
2	*Dicer^+/+^;smMHC^Cre-GFP/+^*	13	10	3	Wild type	Normal
3	*Dicer^lox/lox^;smMHC^+/+^*	7	3	4	Wild type	Normal
4	*Dicer^lox/+^;smMHC^Cre-GFP/+^*	8	4	4	Wild type	Normal
5	*Dicer^lox/+^;smMHC^+/+^*	27	11	16	Wild type	Normal
6	*Dicer^lox/Δ^;smMHC^+/+^*	10	10	0	Heterozygous null	Normal
7*	*Dicer^lox/Δ^;smMHC^Cre-GFP/+^*	13	7	6	Homozygous null	GI distension
8	*Dicer^lox/Δ/+^;smMHC^Cre-GFP/+^*	23	13	10	Mixed	Normal
9	*Dicer^Δ/+^;smMHC^+/+^*	7	5	2	Heterozygous null	Normal
10	*Dicer^Δ/+^;smMHC^Cre-GFP/+^*	21	12	9	Heterozygous null	Normal

*Dicer^lox/Δ^;smMHC^Cre-GFP/+^* was renamed to *smDicer^−/−;Cre-GFP/+^*.

### Knockdown of SMC miRNAs in the *smDicer* mutant mice

**Figure 3:**
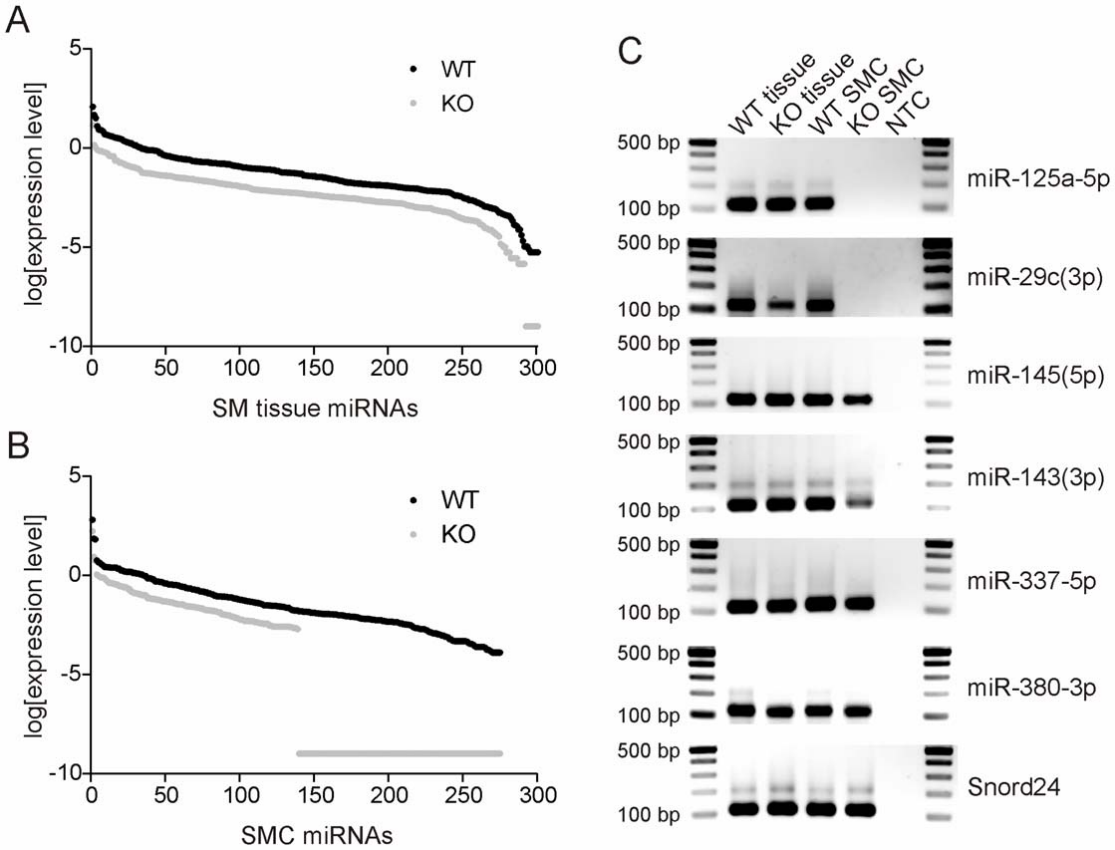
Reduction of miRNAs in the *smDicer* knockout SMCs. The expression level of SMC miRNAs was examined in the small intestinal SM and SMCs from the wild type (WT) control and *smDicer* knockout (KO) mice by qPCR. Expression levels for each miRNA were normalized by an average expression level of the 5 snoRNA genes. Normalized expression levels were converted into a log scale and then plotted on the graph. (**A**) Knockdown of miRNAs in the *smDicer* KO SM. (**B**) Knockdown of miRNAs in the *smDicer* KO SMCs. Note that 87% of the miRNA's expression level was reduced in the *smDicer* KO SM while 99% was reduced in the *smDicer* KO SMCs. (C) Representative gels of qPCR amplicons. miR-125a-5p, miR-29c(3p), miR-145(5p), miR-143(3p), miR-337-5p, and miR-380-3p were analyzed on the gels. snoRNA U24 (Snorrd24) was employed as one of the 5 endogenous genes used for the normalization.

Knockdown of SMC miRNAs in the *smDicer* mutants was examined by qPCR. We previously cloned and identified a SM miRNAome (312 miRNAs) from mouse small intestinal tissue [26]. Extensive expression profiles of the SM miRNAome were made by qPCR from the small intestine tissue and sorted SMCs from the *smDicer* mutants and the wild type controls (see Table S2). Among the 310 SM miRNAs screened by qPCR, 301 and 292 miRNAs were detected in the small intestinal tissue from the wild type control and mutants, respectively (Table 2; details in Table S2). However, expression levels of 268 miRNAs were reduced in the mutant tissue with >2 folds compared to the wild type control (Figure 3A). The 13% median value in the mutant tissue indicated that 87% of the miRNA's expression level was knocked down in the mutant tissue, showing that expression levels of miRNAs were dramatically reduced in the mutant SMCs (Table 2). qPCR detected 275 in the wild type SMCs, but about half of the miRNAs (136 miRNAs) were not detected in the mutant SMCs while 222 miRNAs showed reduction of expression level >2 fold (Figure 3B and Table 2). The 0.1% median value in the mutant SMCs indicated 99.9% of the miRNAs' expression level was knocked down in the mutant SMCs (Table 2). Among the 275 SMC miRNAs, 136 miRNAs including miR-125a-5p and miR-29c(3p) were completely abolished in the mutant SMCs. Expression levels of miR-125a-5p and miR-29c(3p) were greatly reduced in the mutant tissue, but were not detected in the mutant SMCs (Figure 3C and Table S2). However, half of the SMC miRNAs including miR-145(5p) and miR-143(3p) were still detected in the mutant SMCs although expression levels of most of the SMC miRNAs were significantly reduced in the SMCs (Figure 3C and Table S2). This finding suggests that the 136 miRNAs abolished in the mutant SMCs have a shorter life span in comparison to the mature miRNAs, whereas the remaining miRNAs found in the SMCs have a relatively longer life span. Interestingly, 26 miRNAs including miR-337-5p and miR-380-3p were upregulated >2 fold in the mutant SMCs (Figure 3C, Table 2 and Table S2). These miRNAs may be generated by *Dicer*-independent miRNA biogenesis pathways such as Ago2 [40], [41].

**Table 2:** Summary of miRNAs knocked down in *smDicer* KO SM tissue and SMCs.

	SM tissue		Dysregulation in KO			SMC		Dysregulation in KO		
	WT	KO	Median	Down	Up	WT	KO	Median KO	Down	Up
**miRNAs**	301	292	13%	268	4	275	139	0.1%	222	26

Up/down: >2 fold changes.

### Functional changes in the *tunica muscularis* of the *smDicer* mutant mice

**Figure 4:**
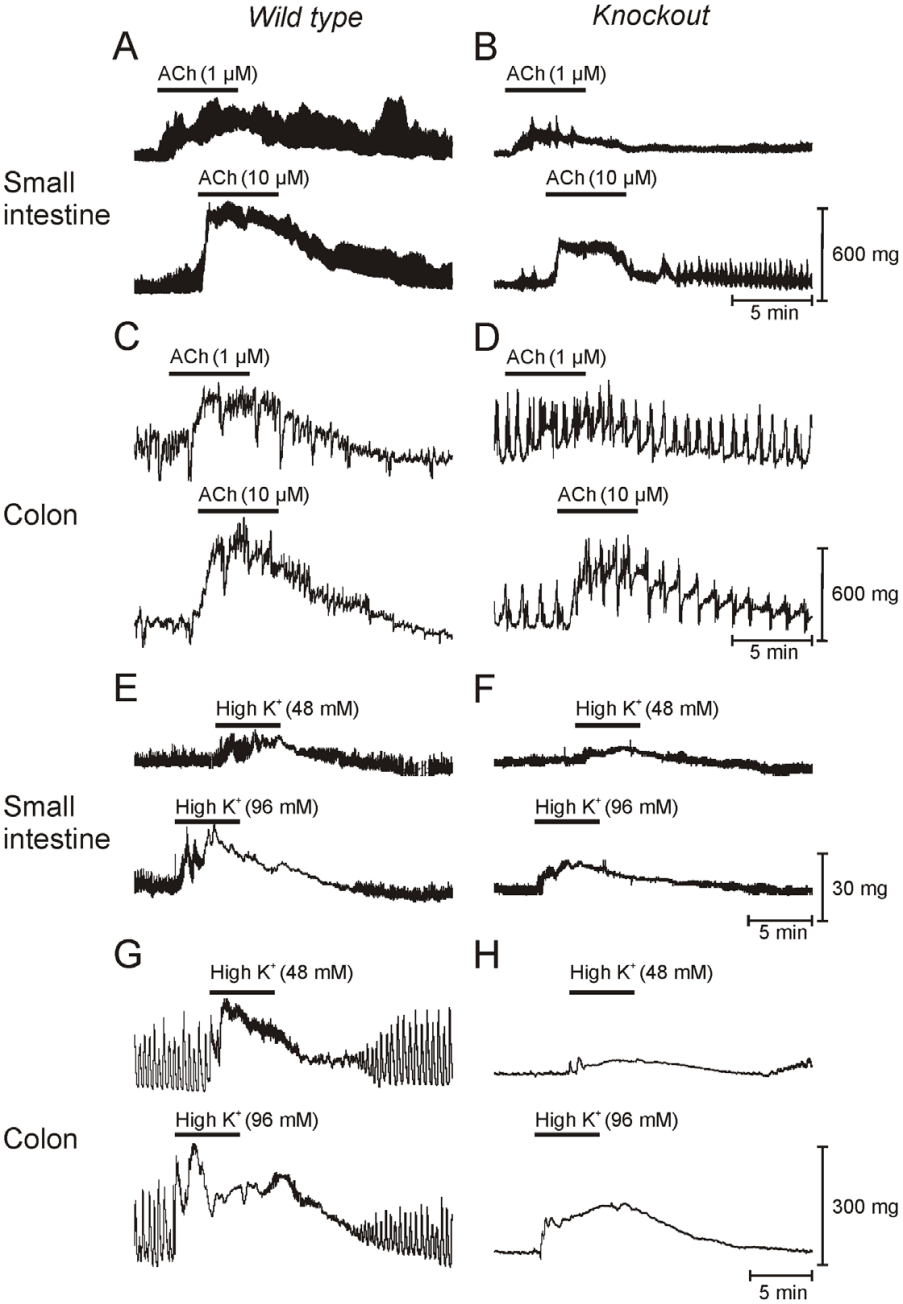
Reduced small intestinal and colonic contractility in the *smDicer* knockout mutants compared to the wild type controls. (**A–D**) The effects of ACh (1 & 10 µM) on the small intestines and colons of control (A, C) and mutant tissues (B, D) respectively (solid bars indicate the time of exposure). (**E–H**) The effect of increasing extracellular [K^+^] (48 & 96 mM) in small intestines and colons of control (E, G) and mutant tissues (F, H). Experiments were preformed in the presence of L-NNA (100 µM) to reduce influences from inhibitory motor nerves. (n = 3)

Isometric force measurements of small intestinal and colonic muscle tissues were performed to characterize functional changes in the *tunica muscularis* of *smDicer* mutants compared to wild type (WT) controls. Spontaneous activity was reduced in the small intestines and colons from the mutant mice compared to those from the WT control mice (Figure 4). In the small intestines from the mutant mice, the amplitude of phasic contractions decreased by approximately 23% (Figure 4B and F) compared to the WT controls (Figure 4A and E). Colonic activity consisted of periodic large amplitude phasic contractions occurring at ∼3 cpm in WT controls (Figure 4C and G) and mutant animals (Figure 4D and H); however, phasic activity was less organized in mutant mice compared to the controls. ACh (1 and 10 µM) enhanced contractions of small intestinal and colonic muscles (Figure 4A and C). Responses to cholinergic stimulation were greatly reduced in mutant mice. Intestinal responses to cholinerigic stimulation were reduced by 31% and 35% to exogenous ACh (Figure 4A and B; 1 & 10 µM, respectively). Colonic responses were reduced by 49% to both 1 and 10 µM ACh (Figure 4C and D). Loss of responsiveness to cholinergic stimulation was not simply due to defects in this specific signaling pathway because contractions elicited by membrane depolarization (e.g. exposure of muscles to elevated extracellular [K^+^]; 48 and 96 mM) were also reduced in the small intestines by 24% and 35% (Figure 4E and F; 48 and 96 mM external K^+^, respectively) while colons of mutant mice by 57% and 29%; Figure 4G and H, 48 and 96 mM extracellular [K^+^], respectively).

### SM genes and transcriptional regulators are dysregulated in the *smDicer* mutant mice

**Figure 5:**
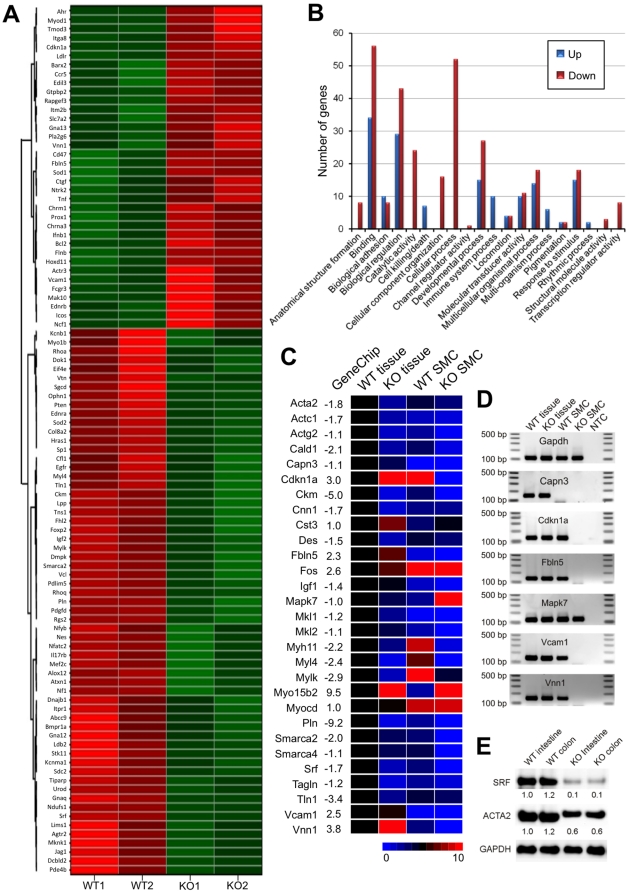
Expression profiles of dysregulated SM genes from the *smDicer* knockout SM tissues and SMCs. (**A**) Gene expression data was obtained using Affymetrix GeneChip Mouse Genome 430 2.0 Array on small RNAs isolated from the small intestinal muscularis of 20-day old wild type (WT) and *smDicer* knockout (KO) mice (n = 2). Among ∼39,000 transcripts, 540 previously known SM genes were chosen for further analysis. Data was filtered using a P-value cutoff of 0.05 for expression and for the same + or − values in the two samples resulting in 97 SM genes (36 upregulated and 61 down-regulated). Clustering dendrograms show the relative expression values according to the following coloring scheme: red = high, white = moderate, green = low. (**B**) Comparison of GO annotation in up- and down-regulated SM genes. (**C**) To confirm the expression profiles from the array, qPCR was performed on cDNAs made from the WT and KO small intestinal SM tissues and SMCs. The expression level of each gene was normalized by an average Ct value of *Gapdh* and *Actb*. Since the WT tissue expressed all the genes tested, the normalized value of the control genes was further normalized by that of the WT tissue. On the heat map, the WT tissue genes were set up as 5 (black) in a range of 0 (lowest) to 10 (highest), and genes of the KO tissue, WT, and KO SMC were expressed relative to that of the WT. GeneChip fold changes are shown along with the gene names. (**D**) Confirmation of qPCR products showing the discrepancy between the tissues and SMCs was analyzed on 2% agarose gels. (**E**) Western blot analysis showing reduction of SRF and SM α-actin (ACTA2) in the *smDicer* KO small intestine and colon. Expression level of each protein was normalized by GAPDH.

SM gene expression in small intestinal SM was analyzed by GeneChip microarrays. The microarrays yielded expression profiles of ∼39,000 transcripts from the *smDicer* KO and WT control mice. The intestinal SM contains SMCs and several other cells including interstitial cells of Cajal (ICC), nerve cells, glial cells, fibroblasts, and cells of hematopoietic origin. To examine gene changes in SMCs, we selected 540 previously identified SM genes collected from the GenBank for further analysis. Data filtering using a P-value cutoff of 0.05 in expression yielded 97 SM genes which were expressed aberrantly in the *smDicer* KO mice in comparison to the WT mice (Figure 5A). Among the dysregulated SM genes, 36 were up-regulated while 61, including *Srf*, were down-regulated in the *smDicer* KO mice (Figure 5A). Interestingly, 35 genes among the dysregulated genes are known to be SRF target genes [12], [42] (see Table S3). There are more SRF target genes (26) among those down-regulated than those (9) up-regulated. The seven genes, *Pln*, *Ckm*, *Tln1*, *Mylk*, *Fhl2*, *Ldb2*, and *Tns1*, which were shown to be the most significantly reduced in the *smDicer* KO mice, are all SRF target genes (see Table S3).

Gene Ontology (GO) annotation comparing up- and down-regulated SM genes revealed that the two groups were different in biological processes ascribed to each gene set (Figure 5B). Immune system processes, cell killing/death, and multi-organism processes were found only in the up-regulated SM genes but not in the down-regulated, indicating that genes involved in these biological processes are over-expressed in the *smDicer* KO SMCs. Conversely, cellular process, catalytic activity, cellular component organization, anatomical structure formation, transcriptional regulator activity, and structural molecule activity appeared only in the down-regulated SM genes, indicating that genes involved in these biological processes are missing or significantly down-regulated in the *smDicer* KO SMCs (Figure 5B). In addition, binding, biological regulation, and developmental processes were greatly increased in the down-regulated SM genes, indicating that genes involved in these biological processes are also greatly down-regulated in the *smDicer* KO SMCs (Figure 5B).

Dysregulated genes were confirmed in SM tissue and SMCs from the *smDicer* KO mice through qPCR. In the *smDicer* KO small intestine tissues, 29 dysregulated genes, including 6 SRF target genes (see Table S3) from the GeneChip data, matched qPCR data (Figure 5C). Expression profiles of all 29 genes tested from the *smDicer* KO tissues in comparison to the WT tissues were consistent between the GeneChip array data and the qPCR data. Furthermore, qPCR expression profiles of the 22 genes from the *smDicer* KO SMCs and WT SMCs were consistent with the GeneChip data (Figure 5C). Seven genes including *Srf*, *Ckm*, *Mkl2*, *Myl4*, *Smarca2*, and *Smarca4* were not detected in the *smDicer* KO SMCs by qPCR (see Figure S1). In addition, expression profiles of the 6 genes obtained by qPCR were much different in SMCs than in tissues (Figure 5D). *Capn3* was not detected in either *smDicer* KO or WT SMCs. *Cdkn1a*, *Fbln5*, *Vcam1*, and *Vnn1*, which were up-regulated in the *smDicer* KO tissue, were not detected in the *smDicer* KO SMCs. Conversely, *MapK7*, which was down-regulated in the *smDicer* KO tissue, was actually up-regulated in the *smDicer* KO SMCs (Figure 5D). Taken together, this qPCR data showed that all 6 SRF target genes (see Table S3) tested were abolished or down-regulated in the *smDicer* KO SMCs. In addition, we validated the reduction of two important proteins, SRF (the master switch for SM contractile genes) and ACTA2 (SM α-actin, a well-known marker for SMCs), in the KO small intestine and colon tissues by Western blot. Consistent with the expression profiles obtained from the microarray and qPCR results, SRF and ACTA2 were significantly reduced by 90% and 40% respectively in the KO tissues (Figure 5E).

We also analyzed miRNAs targeting 58 transcriptional regulators that were dysregulated in the microarray from the *smDicer* KO mice. Among the 58 regulator genes, 15 were up-regulated while 43 were down-regulated in the *smDicer* KO SM (see Table S4). About half of the genes are known to be positive (17), negative (12), or both positive and negative (6) regulators during transcription in the GO categories. Nineteen genes are involved in cell proliferation (8), differentiation (18), or both proliferation and differentiation (7) in the GO categories. Those involved in both show cell-specific proliferation, or differentiation in different cell types. We examined the 19 regulatory genes associated with cell proliferation and differentiation in small intestinal SM tissues and SMCs from the *smDicer* WT and KO mice by PCR. The WT SM tissue expressed 11 regulatory genes detected by PCR while the *smDicer* KO SM tissue expressed 9 genes (see Figure S2 and Table S4). The WT SMCs expressed 6 genes (*Arnt, Hif1a*, *Id4*, *Nkx2-3*, *Pgr*, and *Znrd1*), but 4 genes (*Barx2, Irx5, Neurod4, Pou3f1*, and *Prox1*) were not detected by PCR. However, all of the 6 genes expressed in the WT SMCs were abolished in the *smDicer* KO SMCs (see Figure S2). The 6 regulatory genes are associated with cell proliferation (*Pgr*), differentiation (*Znrd1, Hif1a*, and *Arnt*), or both proliferation and differentiation (*Id4* and *Nkx2-3*) in the GO categories (see Table S4).

### Dysregulated SM genes and transcriptional regulators are potential targets of SM miRNAs

To see if there was a link between the dysregulated SM genes in the *smDicer* mutant mice and the SM miRNAome that we previously cloned and identified from mouse small intestine [26], we retrieved all putative targeting miRNAs using the 97 dysregulated SM genes from miRNA targeting databases PicTar [37] and TargetScan [38], [39] and analyzed how many miRNAs among the targeting miRNAs were found in the SM miRNAome. In the SM miRNAome, we previously grouped the cloned SM miRNAs into two categories: SM common (found in mice and humans) and SM unique miRNAs (found in either mice or humans) [26]. Most targeting miRNAs retrieved from PicTar and TargetScan (see Table S5) were found in the SM miRNAome [26]. Average hit per miRNA from PicTar was significantly higher (4 times higher compared to the non SM miRNAs) in SM common miRNAs (0.59) than in SM unique (0.36) and in non SM miRNAs (0.15) (see Table S5). A similar result was obtained from TargetScan: 0.66 (5 times higher) in SM common, 0.21 in SM unique, and 0.14 in non SM miRNAs (see ).

We also analyzed miRNAs targeting 58 transcriptional regulators that were dysregulated in the microarray from the *smDicer* mutant mice. Among the 58 transcriptional regulators, 15 were up-regulated while 43 were down-regulated (see Table S6). Both PicTar and TargetScan predicted most SM miRNAs (SM common and SM unique) compared to non SM miRNAs. Average hit per miRNA from PicTar was considerably higher (6 times higher compared to the non SM miRNAs) in SM common miRNAs (0.43) than in SM unique (0.22) and in non SM miRNAs (0.07) (see Table S6). A similar result was obtained from TargetScan: 0.62 (5 times higher) in SM common, 0.20 in SM unique, and 0.12 in non SM miRNAs (see Table S6).

This miRNA targeting analysis shows there is a close link between SM common miRNAs and the dysregulated SM genes or transcriptional regulators, suggesting that SM common miRNAs may target the dysregulated SM genes and transcriptional regulators, leading to the phenotypic changes in the *smDicer* mutant SMCs.

## Discussion

### The *smDicer* KO mice implicated in the regulation of SMC phenotype by the SRF-dependent, SMC-phenotypic miRNAs

In this study, we showed that specific KO of *Dicer* in SMCs resulted in a dramatic phenotype in the GI tract. The *smDicer* mutant mice showed significantly thinner longitudinal and circular muscle layers as well as aberrant SMCs in the GI tract (Figure 2). This mutant SMC phenotype is remarkably similar to that of the inducible, SM-specific *Srf* null mice that developed severe dilation of the intestinal tract associated with the thinning of muscle layers [43], [44]. We previously cloned and identified the intestinal SMC miRNAomes from mice and humans, a large number of which are regulated by SRF through CArG boxes found around the SMC miRNA genes [26]. Our SMC-specific *Dicer* KO mice, along with the *Srf* null mice, confirmed that SMC phenotype is modulated, in part, via the SRF-dependent, SMC miRNAs through the SMC miRNA CArG boxes.

### The mutant SMC phenotype and human intestinal obstruction

The extensively dilated intestinal tract associated with impaired SMCs shown in Figure 2 is a typical feature of congenital MMIHS [30] or CIPO [29]. MMIHS is a congenital form of CIPO that causes functional neonatal intestinal obstruction. MMIHS occurs in the GI tract and urinary bladder, and is the most severe form of functional intestinal obstruction in newborns [45]. The cellular and molecular mechanisms underlying this disorder are largely unknown. The major feature of this congenital and usually lethal anomaly is abdominal distension caused by degenerative SMCs [46]. Contractile SM genes were down-regulated in the mutant SMCs (Figure 5A and C), similar to alterations found in MMIHS [47] and the CIPO model mice [44]. Expressions of SM α-actin (*Acta2* in mice), calponin (*Cnn1*), caldesmon (*Cald1*), and desmin (*Des*) that were significantly reduced in MMIHS [47], [48] were also reduced in the mutant SM tissue and isolated SMCs (Figure 5C, E and Figure S1). In addition, reduction of SRF (*Srf* in mice) and smoothelin-A (*Smtnl1*) found in CIPO [43],[44],[49] is also seen in the mutant SM and/or SMCs (for *Srf*, see Figure 5C, E and Figure S1; for *Smtnl1*, see the NCBI GEO array dataset GSE21738 deposited in this study). This similarity suggests that the *smDicer* mutant mice may be a model that could be useful in studies of MMIHS or CIPO, and that MMIHS or CIPO is possibly linked to the defective expression or function of SRF-dependent SMC miRNAs.

### Affected miRNAs from the disruption of *Dicer* in SMCs

miRNAs affected in several different cell- or tissue-specific *Dicer* KO animals are quite different. Abolished miRNAs depend on the cell type, location, and time when *Dicer* is disrupted during the development of the cells. miRNA biogenesis is primarily regulated at the level of gene transcription [50]. Although some transcriptional factors (e.g. HIF1α, MYC, NF-kB, c-Jun, and p53) have been found to regulate the promoter activity of miRNAs [51]–[54], transcriptional regulation of miRNAs are complicated and largely unknown. miRNAs are generated from intergenic regions [probably non-coding RNAs (ncRNAs)], introns, and exons (5′ or 3′ UTR, and CDS). What's even more complicated is the fact that some miRNAs are generated from the antisense of introns and exons. We analyzed SM miRNA transcriptomes that we previously cloned and identified from humans and mice [26]. In mouse miRNAs, 48% of the miRNAs originated from mRNAs, 42% were from known transcripts, and 10% were from ncRNAs. In human miRNAs, 57% of the miRNAs originated from mRNAs, 30% were from unknown transcripts, and 13% were from ncRNAs. Half (50%) of the mouse miRNAs were located within intergenic regions, 41% in introns, and 1% in exons. Interestingly, 8% of the miRNAs were located in either antisense strand of introns or exons, suggesting they are transcribed by their own anti-directional promoters. The origin of human miRNAs showed a similar distribution: 43% in intergenic regions, 48% in introns, 2% in exons, and 7% in antisense-introns. This heterogeneous origin of miRNAs suggests each group of miRNAs with the same origin may be generated and maintained in a different transcriptional pathway. In this study, we demonstrated the expression of SM miRNAs was reduced by 87% in the KO SMCs: 136 miRNAs were not detectable, 222 miRNAs were reduced with >2 fold, and 26 miRNAs including miR-451 were upregulated with >2 fold (Table 2; details in Table S2). Most SM miRNAs were downregulated and Dicer-dependent. However, the differential reduction data suggests that mature miRNAs are differentially reduced and disappear in the developing SMCs after ablation of *Dicer* at E12.5 days when *Cre* is activated in the cells [31]. Differential reduction of miRNAs is consistent in other *Dicer* KO mice [8], [55]. This differential reduction of miRNAs in the KO cells may be due to different turnover and stability rates for each miRNA in multiple biogenesis pathways in different tissues [56], [57]. The 26 upregulated miRNAs identified in this study are also of interest because they may be generated by multiple Dicer-independent pathways. Recent studies showed that miR-451 is indeed processed by Dicer-independent Ago2 [40], [41]. It would be interesting to see if the other upregulated miRNAs are processed by Ago2 or by other Argonaute proteins.

### EGFP specifically expressed in the SMCs as a powerful tool to study GI pathology

We generated SMC-specific *Dicer* null mice that expressed eGFP in a SMC-specific manner (Figure 2). It is critical to purify SMCs from other cells in order to study the changes in gene expression specific to SMCs. GI SM, like other tissues, contains a heterogeneous population of cells. Currently, there are no known specific surface antigens for SMCs that can be used as a strategy for isolating these cells via FACS. We circumvented this problem by using the expression of eGFP which was directed to specific SMC expression via the MHC promoter (*smMHC^Cre-GFP/+^*) mice [31]. We previously showed intestinal SMCs sorted from the mice were differentiated SMCs [26]. These mice display bright green fluorescence in all of the SMCs in the GI tract and also express Cre recombinase in SMCs. We crossed these mice with a mouse we bred (*Dicer^lox/lox^*) to generate SMC-specific *Dicer* null offspring. The expression of eGFP in SMCs allowed for the evaluation of the structure of the *tunica muscularis* via confocal microscopy, FACS, and purification of SMCs for the analysis of gene expression. This transgenic model using SMC-specific eGFP expression provides a powerful new tool to study the function and genetic regulation of SMC phenotypes that may be altered as a result of MMIHS or CIPO.

### Inflammatory infiltrate in the *smDicer* KO colon

We observed atrophy of both the circular and longitudinal muscle layers in the colonic muscularis (Figure 2D). The colonic mucosal layer exhibited inflammation accompanied by an extensive inflammatory infiltrate correlating to the onset of colitis [58] (Figure 2F). It would appear that the inflammatory cell infiltrate is most notable in the lamina propria, submucosa, and muscularis mucosa regions. The predominant cells comprising the inflammatory infiltrate in the dextran sulphate sodium (DSS)–induced colitis animal model are macrophages, neutrophils, and eosinophils [58]. These cell types may be responsible for the extensive inflammatory infiltrate evident in Figure 2F, but in order to precisely identify these cell types, immunohistochemistry would be required. Another notable observation in the inflamed colon from the *smDicer* KO mice is that many cells within the mucosal lining appear apoptotic although confirmation by TUNEL is required for verification. The inflammatory infiltrate might be responsible for the increased expression of the genes involved in immune system processes and cell killing/death in the *smDicer* KO mice (Figure 5B). In addition, GI SMCs from patients with ulcerative colitis, an idiopathic inflammatory disease of the colon, showed an abnormal motor dysfunction in the colonic circular muscle while the muscularis propria contained increased pro-inflammatory cytokine signals [59]. These observations in the colonic colitis are similar to the abnormalities (inflammatory infiltrate, increased immune system and apoptotic activities, and reduced contractility) in the inflamed colon obtained from the *smDicer* KO mice in this study. Taken together, our data suggest that the abnormal SMCs may trigger inflammatory infiltrate while normal SMCs should have some mechanisms to suppress or bypass the pro-inflammatory cytokine signals.

### A Network of interaction among SRF, SM miRNAs, transcriptional factors, and SM genes

**Figure 6:**
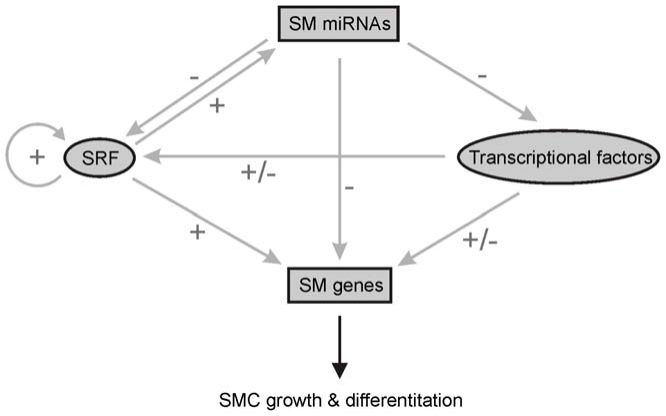
Model of the regulation of SM genes by SM miRNAs, SRF, and transcriptional factors. SM genes are regulated by multiple pathways that fine tune SMC growth and differentiation. 1) SM genes are directly and positively regulated by SRF; SM genes are SRF targets that are transcriptionally turned on by SRF [15]. 2) SM genes are directly and negatively affected by SM miRNAs; SM miRNAs target SM genes (see Table S5). 3) SM genes are indirectly and negatively controlled by SRF through SM miRNAs; we previously found that a large number of SM miRNAs, such as miR-143/miR-145 and miR-199a/miR-214, are SRF targets [26], and SM miRNAs target SM genes (see Table S5). 4) SM genes are directly and positively/negatively affected by transcriptional factors; in the *smDicer-GFP* mice, transcriptional factors up or down-regulated are positive and/or negative regulators of transcription for SM genes (see Table S4). 5) SM genes are indirectly and positively/negatively controlled by SM miRNAs through transcriptional factors; transcriptional factors are potential SM miRNA targets (see Table S6) and repressed transcriptional factors regulate the expression of SM genes. 6) SM genes are indirectly and positively/negatively affected by SRF through SM miRNAs and transcriptional factors; SRF turns on SRF-dependent SM miRNAs, which target transcriptional factors. Repressed transcriptional factors regulate the expression of SM genes.

We showed that aberrantly expressed SM genes and transcriptional regulators are potential targets of SM miRNAs (see Tables S5 and S6). Collectively, our studies regarding the SM miRNAome analyzed with the miRNA expression and targeting analyses lead us to propose a model that SM genes are regulated by multiple pathways to control SMC growth and differentiation (Figure 6). Expression of SM genes is affected by positive and negative regulation in a network of SM miRNAs, SRF, and transcriptional factors. SM miRNAs negatively regulate SRF, SM genes, and transcriptional factors at the post-transcriptional level by binding to their target transcripts. Transcriptional factors positively or negatively regulate SRF and SM genes at the transcriptional level by binding to their regulatory sites, while SRF positively regulates SM miRNAs, SM genes, and SRF itself at the transcriptional level by binding to their CArG or CArG-like boxes. This model presents a network of interaction among SRF, SM miRNAs, transcriptional factors, and SM genes and offers the unique opportunity to study the regulation of the SMC phenotype in other pathological contexts.

## Supporting Information

Text S1 Extended Materials and Methods.
(DOC)

Figure S1 **Confirmation of qPCR products showing the discrepancy between the smooth muscle tissues and sorted SMCs from the wild type control and the** ***smDicer*** **knockout mice.** qPCR products from Figure 5C were analyzed on 2% agarose gels. *Actb* was used as an endogenous control.
(TIF)

Figure S2 **Expression of 11 transcriptional regulators in the smooth muscle tissues and sorted SMCs from the wild type control and the** ***smDicer*** **knockout mice.** PCR products were analyzed on 2% agarose gels. *Actb* was used as an endogenous control.
(TIF)

Table S1 Oligonucleotides used in this study.
(DOC)

Table S2 Reduction of miRNAs in small intestine tissue and SMCs from the *smDicer* knockout mice.
(XLS)

Table S3 A list of SM genes dysregulated in the *smDicer* knockout mice.
(XLS)

Table S4 A list of transcriptional regulators dysregulated in the *smDicer* knockout mice.
(XLS)

Table S5 A list of miRNAs targeting SM genes.
(XLS)

Table S6 A list of miRNAs targeting transcriptional regulators.
(XLS)
