# Supplementary material for: MicroRNAs Dynamically Remodel Gastrointestinal Smooth Muscle Cells
Source: PLoS One. 2011 Apr 14;6(4):e18628. doi: 10.1371/journal.pone.0018628 (PMC3077387; doi:10.1371/journal.pone.0018628)
Supplement: Text S1 — Extended Materials and Methods. (DOC) [file pone.0018628.s001.doc]

**Extended Materials and Methods**

**Generation of *smDicer* mutant mice**

A SMC-specific *Dicer* knockout (KO) *Dicer^lox/lox^;smMHC/Cre/eGFP* (*smDicer^-/-;Cre-GFP/+^*) mouse line was generated by cross-breeding a *smMHC/Cre/eGFP* (*smMHC^Cre-GFP/+^)* male mouse (gift from Kotlikoff M.I. at Cornell University) [1] and a *D*icer^lox/lox^ female homozygote mouse (The Jackson Laboratory) according to the National Institutes of Health (NIH) Guide for the Care and Use of Laboratory Animals and the American Physiological Society’s ‘Guiding Principles in the Care and Use of Animals’. All procedures (Protocol 00097) used in generating and analyzing the transgenic mice were approved by the Institutional Animal Care and Use Committee at the University of Nevada, Reno. The offspring mice were genotyped with sets of primers: Cre-2/Cre-2r for the *Cre* gene, eGFP-2/eGFP-2r for the *eGFP* gene, Dicer-F2/Dicer-R2 for the loxP inserted and WT loci, and Dicer-F2/Dicer-Del2 for the *Dicer* deletion (∆) (see Table S1) through genotyping PCR. PCR products amplified with each set of primers were analyzed on 1.5% agarose gels and then sequenced for confirmation. Transgenic mice and wild type (WT) mice (siblings of transgenic mice) were maintained until used for the following experiments.

**Tissue preparation**

The mutant *smDicer^-/-;Cre-GFP/+^*, WT *smMHC^Cre-GFP/+^,* or C57BL6 mice were anesthetized by isoflurane (AErrane) inhalation and killed by decapitation at the age of ~3 weeks. Multiple tissues (brain, heart, kidney, liver, lung, spleen, bladder, trachea, vein, testis, ovary, uterus, stomach, small intestine, cecum, and colon) were dissected from the mice. The gastrointestinal (GI) smooth muscle tissues (stomach, small intestine, cecum, and colon) were stripped free of mucosa and submucosal plexus. The multiple tissues were used for the isolation of total RNAs. Some of the small intestine and colon muscularis tissues were used for purification of smooth muscle cells (SMCs) using flow cytometry and fluorescence-activated cell sorter (FACS). Small intestinal tissues from ~3-weeks-old C57BL6 mice were used for organ culture. The GI tissues were used for microscopic histological analysis or for mechanical response studies.

**Isolation of total and small RNAs from tissues and cells**

Total RNAs and/or small RNAs were isolated from multiple tissues and sorted SMCs from the *smDicer^-/-;Cre-GFP/+^* and the WT control mice using the mirVanaTM miRNA isolation kit (Ambion) as previously described [2]. Briefly, 50-250 mg of tissue or 50,000-200,000 cells (eGFP-positive cell populations sorted by FACS) was homogenized in the Lysis/Binding buffer. A 1/10 volume of miRNA Homogenate Additive was added and incubated on ice for 10 min. Total RNA was first extracted by adding an equal volume of Acid-Phenol:Chloroform. Small RNA was then extracted from the total RNA using a filter cartridge with 100 μl of preheated (95 °C) elution solution. The remaining total RNA was also isolated using another filter cartridge with 100 μl of preheated (95 °C) elution solution. The concentrations (ng/μl) of total RNAs and small RNAs were measured using an ND-1000 spectrophotometer (NanoDrop Technologies).

**RT-PCR and qPCR detection of messenger and small RNAs**

Preparation of the cDNA libraries from the total RNAs isolated from multiple tissues and sorted SMCs (pooled from 4 mice) obtained by FACS from *smDicer^-/-;Cre-GFP/+^* or the WT control mice, as well as qPCR analysis on cDNAs, were performed as previously described [3]. All primers used for RT-PCR and qPCR are shown in Table S1. For qPCR on cDNAs, the expression level (average Ct) of each gene for WT and KO mice was normalized to an average Ct of *Gapdh* and *Actb*. The WT tissue expressed all the genes tested. To see changes in expression of the genes in the KO tissues, the WT SMCs and the KO SMCs were further normalized than the initially normalized values of the WT tissue. On the heat map, the WT tissue genes were set up as 5 (black) in a range of 0 (lowest) to 10 (highest). Genes of the KO tissue, WT, and KO SMCs were expressed relative to that of the WT. RT-PCR was also performed to confirm the purity of sorted SMC populations using gene specific primers for *Gapdh*, *Ncam1* (neuronal cells), *Kit* (ICC), *Ptprc* (Cd45, hematopoietic cells including macrophages), *Cma1* (mast cells), and *Myh11* (smMHC, SMC) (see Table S1) as described previously [4]. Preparation of the small RNA cDNA (srcDNA) libraries from the small RNAs isolated from small intestine tissues and SMCs sorted by FACS, as well as qPCR analysis on srcDNAs, were performed as previously described [5]. All 301 SMC miRNA primers and the five snoRNAs endogenous control genes (genes (*U1A*, *U24*, *U43*, *U58A*, and *snoR-202*) used in this study are shown in Table S1. qPCRs were performed with each of the SMC miRNA primers with different annealing temperatures (Tm) as indicated in the table. The expression level of each miRNA was normalized by an average Ct of the five control snoRNA genes, and then the normalized values were converted to normalized values in Excel and on a graph. All RT-PCR and qPCR products were analyzed on 1.5% agarose gels. Most of the genes of the products were confirmed by direct sequencing at the Nevada Genomic Center (Reno) following a column purification using QIAquick Spin kit (QIAGEN).

**Western blot analysis**

For protein expression analysis, Western blotting was performed as previously described [6]. Briefly, 20 μg of total proteins from the small intestine or colon smooth muscle tissue lysates from the *smDicer^-/-;Cre-GFP/+^* and the WT control mice were separated on 10% SDS-polyacrylamide gels and transferred to Trans-Blot Transfer Medium (Pure Nitrocellulose Membrane, Bio-Rad, Hercules). The membranes were blocked with 5% non-fat milk and incubated with a mouse anti-smooth muscle α-actin (ACTA2) (1:1500) (Dako), a rabbit anti-SRF (1:500) (Santa Cruz Biotechnology), or a mouse anti-GAPDH antibody (1:1500) (Abcam). The protein–antibody complex was detected following incubation with the horseradish peroxidase-conjugated secondary antibodies (1:50 000) (Chemicon) using an enhanced chemiluminescence method (ECL Advantage, Amersham Bioscience). The protein bands were captured using a CCD-camera system (EC3 410 Imaging System, UVP Laboratory Products) and analyzed with VisionWorksLS software (Version 6.8, UVP Laboratory Products). The expression level of ACTA2 and SRF was normalized to the ratio of ACTA2 and SRF area density divided by GAPDH area density. For quantitative measurements, the values of relative expression for ACTA2 and SRF in WT colon, KO small intestine, and KO colon were compared to the expression in WT small intestine (set to 1.0).

**Confocal microscopy and histological analysis**

Enhanced green fluorescent protein (eGFP) fluorescence microscopic analysis was performed as previously described [4]. The small intestine, cecum, and colon were pinned to the Sylgard elastomer (Dow Corning) floor of a dissecting dish and stretched to 110% of the resting length and width. The mucosa and submucosa from the tissues were removed by sharp dissection. Muscularis tissues were fixed in paraformaledehyde (PFA, 4%, w/v) at 4 ^o^C for 30 min. Following fixation, tissues were subsequently washed with 0.01M phosphate buffered saline (PBS, pH 7.4) at 4 ^o^C for several hours and soaked overnight to completely remove the fixative. Fixed tissues were dehydrated in graded sucrose solutions (5%, 10%, 15% and 20% w/v in PBS at 4 ^o^C, 1 hour at each concentration). Tissues were incubated overnight in 20% sucrose in PBS at 4 ^o^C before being embedded in a 1:1 solution of Tissue-Tek (Miles) and 20% sucrose in PBS, and then frozen in liquid nitrogen. Cryostat sections were cut at a 7 μm thickness using a cryostat (CM3050, Leica), air dried for 30 min, and washed in PBS for 30 min. Fluorescent images of section and mount tissues were made on a confocal microscope (LSM 510, Zeiss). Fluorescent images of single SMCs isolated from the intestinal muscularis (see Flow cytometry and fluorescence-activated cell sorting) were captured. For histological analysis, colon tissues from the *smDicer^-/-;Cre-GFP/+^* and the WT control mice were dehydrated, embedded in paraffin, cut into 6 μm-thick coronal sections, rehydrated, and stained with hematoxylin and eosin (H & E). Images were collected using the confocal microscope (Zeiss).

**Flow cytometry and fluorescence-activated cell sorting**

Small intestinal and colonic muscularis dissected from the 3-week old *smDicer^-/-;Cre-GFP/+^* mice and the WT control mice were digested by collagenase (Worthington Biochemical Corporation), triturated through a series of three blunt pipettes, and filtered through a polyester filter with 100 μm mesh size (Partec) to obtain single-cell suspensions as previously described [4]. Dispersed cells were suspended in ~450 μl of a staining buffer [flow cytometry sheath solution, diluted from 8× stock solution (BioSure) with 1% FBS and 0.09% sodium azide] and labeled with Hoechst 33258 (~10 nM). After ~30 min of incubation with the Hoechst stain, the cell suspensions were ready for sorting. Cells were analyzed and sorted (130 μm nozzle, 12 psi) on a Becton Dickinson FACSAria II (Becton Dickinson) equipped with 355 nm, 488 nm and 640 nm lasers with detectors for Hoechst emission (430/50 nm) using the UV laser, and GFP emission (515/30 nm) using the blue laser. The FACSAria has a total of 11 fluorescence detectors but only the listed detectors were used for the studies. FlowJo software (Tree Star) was used to analyze the data. Sorted cells were used for the isolation of total and small RNAs.

**GeneChip arrays for messenger RNAs**

Mouse mRNA microarrays were performed on GeneChip® Mouse Genome 430 2.0 Arrays (Affymetrix) covering 39,000 mouse gene transcripts and variants. Total RNAs isolated from the small intestinal smooth muscle tissues free from mucosa and submucosa of 20-day old KO female *smDicer^-/-;Cre-GFP/+^* mice and WT control mice (2 each) were used. Approximately 2.5 μg of total RNA was treated with a recombinant DNase I using DNA-free DNase Treatment & Removal Reagents (Ambion) to eliminate genomic DNA contamination according to the manufacturer’s instructions except for the addition of an RNase inhibitor, RNaseOUT (Invitrogen), to protect the total RNAs from RNases. After purifying the total RNAs, we analyzed them on a 2100 Bioanalyzer (Agilent Technologies). Hybridization, cDNA labeling, and scanning were performed at the Nevada Genomic Center (Reno). Briefly, approximately 2 μg of the purified total RNAs was used for the first-strand cDNA synthesis and converted into single-stranded cDNAs by Superscript III followed by a second-strand cDNA synthesis. The double-stranded cDNAs were then purified using a GeneChip Sample Cleanup Module Kit (Affymetrix) and converted into biotin-labeled cRNAs using GeneChip Expression 3’-Amplification Reagents (Affymetrix) for *in vitro* transcription (IVT) labeling. Each hybridized Affymetrix GeneChip® array was separately scanned with a GeneChip Scanner 3000 (Affymetrix). Array data was analyzed by Phalanx Biotech Group (Palo Alto, CA) using Omicsoft Array Studio software (Omicsoft Corporation) and normalized using the RMA algorithm. Differential expression P-values were calculated using the mean value of two biological replicate chips for each sample. Among ~39,000 transcripts, 540 previously known SM genes collected from the Entrez Gene at the National Center for Biotechnology Information (NCBI) [7] were chosen for further analysis. Data considered for hierarchical clustering were filtered using a P-value cutoff of 0.05. Clustering dendrograms show relative expression values according to the following coloring scheme: red=high, white=moderate, green=low. Gene ontology (GO) was further analyzed using Omicsoft Array Studio (Omicsoft Corporation) to evaluate the GO enrichment of the dysregulated SM genes and the transcription regulators. The analysis counted the number of genes that fell into each GO category based on the current Phalanx Biotech annotation. GO categories between the up- and down-regulated SM genes were compared. Analyzed array datasets were deposited at the NCBI Gene Expression Omnibus (GEO) (GSE21738).

**Analysis of mechanical response**

Small intestine and proximal colonic tissues (10 mm) from *smDicer^-/-;Cre-GFP/+^* and WT control mice were fixed at one end and attached to a Fort 10 isometric force transducer (WPI). Isometric force was measured as previously described [8]. Tissue strips were mounted in organ baths with one end tied by thread to a fixed hook and the other to a tension transducer. Tissues were immersed in 10 ml tissue baths containing oxygenated KRB solution maintained at 37°C ± 0.5°C. A basal tension of 300 mg was applied. All experiments were performed in the presence of L-NNA (100 μM) to eliminate the possible contribution from nitrergic nerve activity. For contractile studies, the mechanical signals were digitized and recorded to a personal computer running Acknowledge software (BIOPAC Systems) with a computerized data acquisition and analysis system (MP100; BIOPAC Systems).

**Analysis of targeting microRNAs**

Potential targeting miRNAs for 97 dysregulated SM genes and 58 dysregulated transcription regulators were retrieved and analyzed from two well-established target prediction programs: PicTar [9] and TargetScan (release 5.1) [10,11]. All PicTar miRNAs for each gene were retrieved. However, targeting miRNAs for each gene on TargetScan were retrieved only from conserved sites for miRNA families broadly conserved among vertebrates (8mer and 7mer-m8 of conserved and poorly conserved sites) to eliminate false-positive targets. Genechip array data (fold change and P value) and expression profiles (SM-common, SM-unique, and non-SM miRNAs) were shown along with PicTar and TargetScan miRNAs. Total hit miRNAs and average miRNAs per gene in SM-common, SM-unique, and non-SM miRNAs were calculated from PicTar and TargetScan miRNAs.

**References**

1. Xin HB, Deng KY, Rishniw M, Ji G, Kotlikoff MI (2002) Smooth muscle expression of Cre recombinase and eGFP in transgenic mice. Physiol Genomics 10: 211-215.

2. Ro S, Song R, Park C, Zheng H, Sanders KM et al. (2007) Cloning and expression profiling of small RNAs expressed in the mouse ovary. RNA 13: 2366-2380.

3. Ro S, Hwang SJ, Muto M, Jewett WK, Spencer NJ (2006) Anatomic modifications in the enteric nervous system of piebald mice and physiological consequences to colonic motor activity. Am J Physiol Gastrointest Liver Physiol 290: G710-G718.

4. Ro S, Park C, Jin J, Zheng H, Blair P et al. (2009) A Model to Study the Phenotypic Changes of Interstitial Cells of Cajal in Gastrointestinal Diseases. Gastroenterology 138: 1068-1078.

5. Ro S, Park C, Jin JL, Sanders KM, Yan W (2006) A PCR-based method for detection and quantification of small RNAs. Biochem Biophys Res Commun 351: 756-763.

6. Park C, Hennig GW, Sanders KM, Cho JH, Hatton WJ et al. (2011) SRF-dependent microRNAs regulate gastrointestinal smooth muscle cell phenotypes. Gastroenterology, in print.

7. Maglott D, Ostell J, Pruitt KD, Tatusova T (2007) Entrez Gene: gene-centered information at NCBI. Nucleic Acids Res 35: D26-D31.

8. Ward SM, Beckett EAH, Wang XY, Baker F, Khoyi M et al. (2000) Interstitial cells of cajal mediate cholinergic neurotransmission from enteric motor neurons. J Neurosci 20: 1393-1403.

9. Krek A, Grun D, Poy MN, Wolf R, Rosenberg L et al. (2005) Combinatorial microRNA target predictions. Nat Genet 37: 495-500.

10. Friedman RC, Farh KKH, Burge CB, Bartel DP (2009) Most mammalian mRNAs are conserved targets of microRNAs. Genome Res 19: 92-105.

11. Grimson A, Farh KKH, Johnston WK, Garrett-Engele P, Lim LP et al. (2007) MicroRNA targeting specificity in mammals: Determinants beyond seed pairing. Mol Cell 27: 91-105.
