# Supplementary figures and images for: MicroRNAs Dynamically Remodel Gastrointestinal Smooth Muscle Cells
Source: PLoS One. 2011 Apr 14;6(4):e18628. doi: 10.1371/journal.pone.0018628 (PMC3077387; doi:10.1371/journal.pone.0018628)

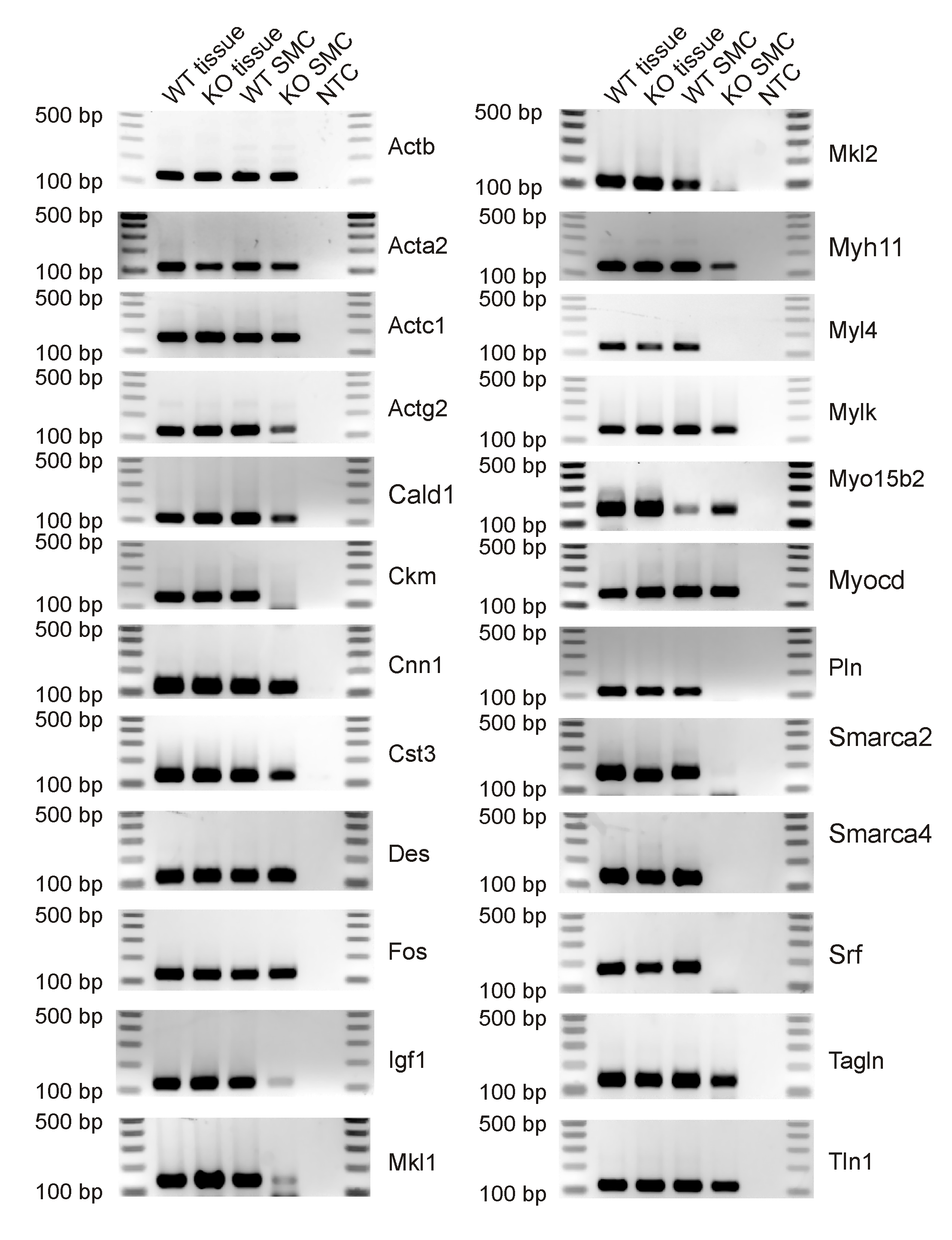

Supplement: Figure S1 — Confirmation of qPCR products showing the discrepancy between the smooth muscle tissues and sorted SMCs from the wild type control and the smDicer knockout mice. qPCR products from Figure 5C were analyzed on 2% agarose gels. Actb was used as an endogenous control. (TIF) [file pone.0018628.s002.tif]

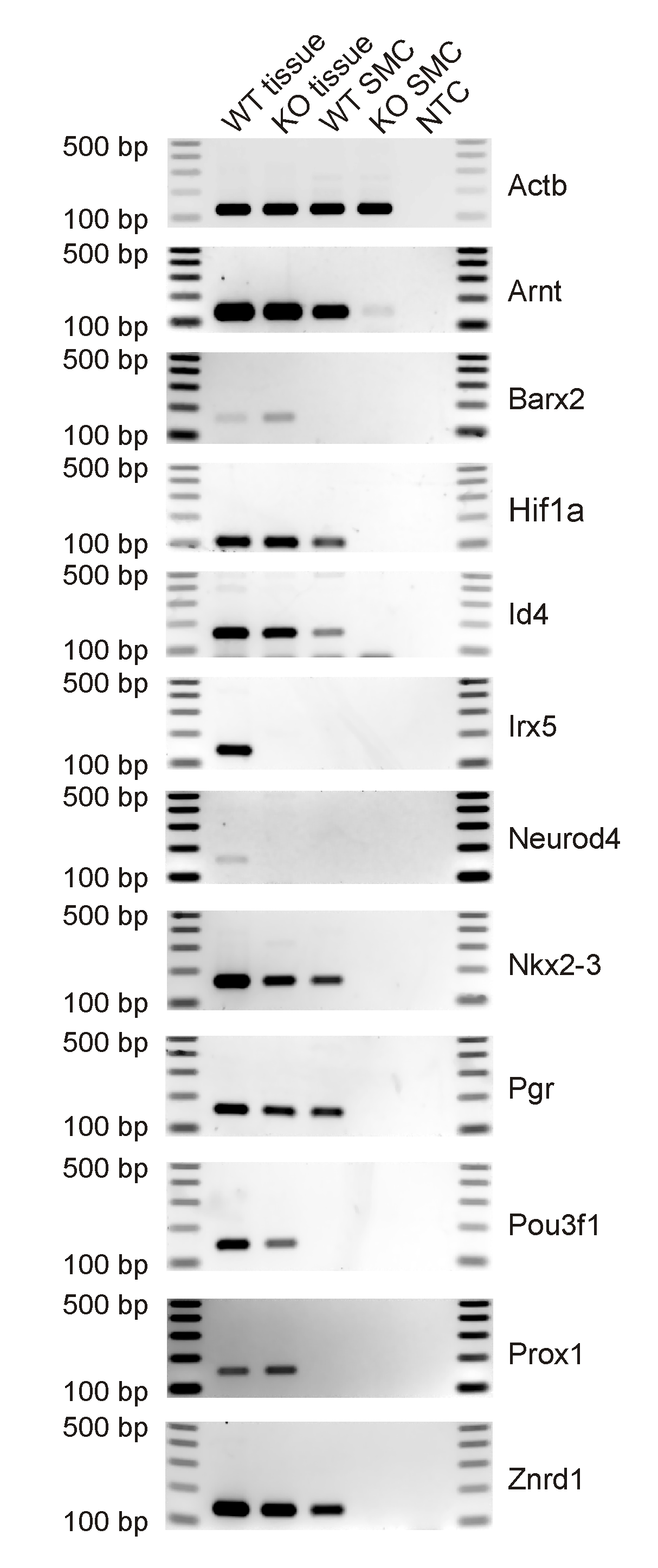

Supplement: Figure S2 — Expression of 11 transcriptional regulators in the smooth muscle tissues and sorted SMCs from the wild type control and the smDicer knockout mice. PCR products were analyzed on 2% agarose gels. Actb was used as an endogenous control. (TIF) [file pone.0018628.s003.tif]
