# Supplementary material for: MicroRNAs Dynamically Remodel Gastrointestinal Smooth Muscle Cells
Source: PLoS One. 2011 Apr 14;6(4):e18628. doi: 10.1371/journal.pone.0018628 (PMC3077387; doi:10.1371/journal.pone.0018628)
Supplement: Table S1 — Oligonucleotides used in this study. (DOC) [file pone.0018628.s004.doc]

**Table S1.** Oligonucleotides used in this study

^1^Size of WT/WT(lox insertion) Dicer loci

^2^ Size of WT/KO(deletion, ∆) Dicer loci

^3^ Size of WT/KO(deletion, ∆) Dicer cDNA

| **Name** | **Sequence**  **(5’ to 3’)** | **Tm**  **(^o^C)** | **Gene** | **Size(bp)**  **c/gDNA** | **Usage** |
| --- | --- | --- | --- | --- | --- |
| Cre-2 | GCATGGTGCAAGTTGAATAACCGG | 57 | Cre | 146 | Genotyping |
| Cre-2r | GACCGACGATGAAGCATGTTTAGC | 57 | Cre |  | Genotyping |
| eGFP-2 | CTGACCCTGAAGTTCATCTGCAC | 57 | eGFP | 134 | Genotyping |
| eGFP-2r | GACTTGAAGAAGTCGTGCTGCTTC | 57 | eGFP |  | Genotyping |
| Dicer-F2 | GTCTCCATCCATTGCTGGAAACTC | 57 | Dicer1 | 262/242^1^ | Genotyping |
| Dicer-R2 | CTAAGAAAGAATTGTGTGCTAGGGCAC | 58 | Dicer1 |  | Genotyping |
| Dicer-Del2 | GCTTGGAGATCAGGCAGCATGG | 59 | Dicer1 | 1261/417^2^ | Genotyping |
| mHMBS-1 | CCTGAAGGATGTGCCTACCATAC | 57 | Hmbs | 125/1087 | RT-PCR/qPCR |
| mHMBS-1r | GCAAGGTTTCCAGGGTCTTTCC | 57 | Hmbs |  | RT-PCR/qPCR |
| mSMH-2 | CTTTTCCGATGGATTCTCAGCCGTG | 59 | Myh11 | 137/979 | RT-PCR/qPCR |
| SMH-2r | GTTGATGCACAGCTGCTCGAAGG | 59 | Myh11 |  | RT-PCR/qPCR |
| Dicer-2 | CTCCTACCACTACAACACTATCACTG | 58 | Dicer1 | 417/146^3^ | RT-PCR |
| Dicer-4r | CTCCAGAGACATCCCACTGTCC | 59 | Dicer1 |  | RT-PCR |
| Gap-2 | TCCTGCACCACCAACTGCTTAGC | 59 | Gapdh | 110/244 | RT-PCR/qPCR |
| Gap-2r | GTCTTCTGGGTGGCAGTGATGG | 59 | Gapdh |  | RT-PCR/qPCR |
| Actb-1 | TTCTACAATGAGCTGCGTGTGGC | 57 | Actb | 124/578 | RT-PCR/qPCR |
| Actb-1r | CTGGGGTGTTGAAGGTCTCAAAC | 57 | Actb |  | RT-PCR/qPCR |
| Ncam1-1 | GAACCTGATCAAGCAGGATGACG | 57 | Ncam1 | 155/3464 | RT-PCR |
| Ncam1-1r | CTTCATACTCTGCGTTCCAGTCC | 57 | Ncam1 |  | RT-PCR |
| Kit-2 | GGATCAGCAAATGTCACAACAACCTTG | 58 | Kit | 158/5512 | RT-PCR |
| Kit-2r | CAAACCCGAGCACCAGCAGTGGATA | 61 | Kit |  | RT-PCR |
| CD-45-2 | GATGTCAGTTGGACAACCTTCGTGC | 59 | Ptprc | 141/3384 | RT-PCR |
| CD-45-2r | CCACAACTAGGCTTAGGCGTTTCTG | 59 | Ptprc |  | RT-PCR |
| Cma1-1 | CTTTGTGCTGACTGCAGCTCACTG | 59 | Cma1 | 159/1160 | RT-PCR |
| Cma1-1r | CATGATGTCGTGGACAACCAAATTCTC | 59 | Cma1 |  | RT-PCR |
| Acta2-1 | CAGAGACTCTCTTCCAGCCATCTTTC | 60 | Acta2 | 119/1533 | RT-PCR/qPCR |
| Acta2-1r | CGTTGTTAGCATAGAGATCCTTCCTG | 58 | Acta2 |  | RT-PCR/qPCR |
| Actc1-1 | CTTTGGTGTGTGACAATGGCTCCG | 59 | Actc1 | 160/1449 | RT-PCR/qPCR |
| Actc1-1r | CTCTGGGCTTCATCACCTACATAG | 57 | Actc1 |  | RT-PCR/qPCR |
| Actg2-1 | GCTCTGGTATTTCTGCCAAAGACAC | 58 | Actg2 | 123/8825 | RT-PCR/qPCR |
| Actg2-1r | GTGGTCTCTTCTTCACACATGGTG | 57 | Actg2 |  | RT-PCR/qPCR |
| Cald1-1 | GAAAGGCTGCGGCAGAAGCAAGA | 59 | Cald1 | 108/3791 | RT-PCR/qPCR |
| Cald1-1 | GGAGGCTGGCTTAGACTCTTCATC | 59 | Cald1 |  | RT-PCR/qPCR |
| Capn3-1 | GACCTGGACGGTGTCTGTAAACG | 59 | Capn3 | 140/2958 | RT-PCR/qPCR |
| Capn3-1r | GAGTCCTCAGGGTCATCGTCTTC | 59 | Capn3 |  | RT-PCR/qPCR |
| Cdkn-1 | CTGTCTTGCACTCTGGTGTCTG | 57 | Cdkn1a | 126/632 | RT-PCR/qPCR |
| Cdkn-1r | GAAGACCAATCTGCGCTTGGAG | 57 | Cdkn1a |  | RT-PCR/qPCR |
| Ckm-1 | CAGCTCATTGATGACCACTTCCTG | 57 | Ckm | 138/2686 | RT-PCR/qPCR |
| Ckm-1r | CTCGTTCACCCACACAAGGAAG | 57 | Ckm |  | RT-PCR/qPCR |
| Cnn1-1 | GGGAACAACTTCATGGATGGCCTC | 59 | Cnn1 | 114/3157 | RT-PCR/qPCR |
| Cnn1-1 | CTGGTGCCAGTTCTGAGTTGACTC | 59 | Cnn1 |  | RT-PCR/qPCR |
| Cst3-1 | CATACAGGTGGTGAGAGCTCGTAAG | 59 | Cst3 | 124/2347 | RT-PCR/qPCR |
| Cst3-1r | GGTCATGGAAAGGACAGTCAGTC | 57 | Cst3 |  | RT-PCR/qPCR |
| Des-1 | CGAGTACCAGGACCTGCTCAATG | 59 | Des | 116/1752 | RT-PCR/qPCR |
| Des-1r | CAGAGAAGGTCTGGATAGGAAGGTTG | 60 | Des |  | RT-PCR/qPCR |
| Fbln-1 | CCTGCTGACATCTTCCAGATGC | 57 | Fbln5 | 135/6487 | RT-PCR/qPCR |
| Fbln-1r | CATCACCAGGGTGGCACTGATAG | 59 | Fbln5 |  | RT-PCR/qPCR |
| Fos-1 | GACAGCCTTTCCTACTACCATTCC | 57 | Fos | 124/878 | RT-PCR/qPCR |
| Fos-1r | CTGTCACCGTGGGGATAAAGTTG | 57 | Fos |  | RT-PCR/qPCR |
| Igf1-1 | GCTGGTGGATGCTCTTCAGTTC | 57 | Igf1 | 117/48851 | RT-PCR/qPCR |
| Igf1-1r | GCAACACTCATCCACAATGCCTG | 57 | Igf1 |  | RT-PCR/qPCR |
| Mapk7-1 | GGGAGCTGAAGATCCTCAAACAC | 57 | Mapk7 | 119/1337 | RT-PCR/qPCR |
| Mapk7-1r | CCATGAGGTCCAGTACCACATAG | 57 | Mapk7 |  | RT-PCR/qPCR |
| Mkl1-2 | GTTATGAAGAGACTGTGACCCAGC | 57 | Mkl1 | 138/2033 | RT-PCR/qPCR |
| Mkl1-2r | CTGGTAGGGATGGTGGCTCTTTG | 57 | Mkl1 |  | RT-PCR/qPCR |
| Mkl2-2 | GATGGACGACCTCTTCGACATC | 57 | Mkl2 | 115/2597 | RT-PCR/qPCR |
| Mkl2-2r | CATTGTGGTGATGCTGGCTGTC | 57 | Mkl2 |  | RT-PCR/qPCR |
| Myl4-1 | CTCTGCCTTTGACCCAAAGAGTG | 57 | Myl4 | 124/6499 | RT-PCR/qPCR |
| Myl4-1r | CTGCCCGTAGGTGATCTTCATC | 57 | Myl4 |  | RT-PCR/qPCR |
| Mylk-1 | GCTCCTGAAGTGATCAACTATGAGC | 58 | Mylk | 142/7287 | RT-PCR/qPCR |
| Mylk-1r | CTGAAGTGACGTTGGCTAAGGTC | 57 | Mylk |  | RT-PCR/qPCR |
| Myo15b2-1 | GGACGAGCTGAGTCTGCTCTATG | 59 | Myo15b2 | 158/1620 | RT-PCR/qPCR |
| Myo15b2-1r | CAAAGAAGCCTGTGAAGAGGCTG | 57 | Myo15b2 |  | RT-PCR/qPCR |
| Myo-2 | CACTCCTGGCTCAGAAAGTGACAAG | 59 | Myocd | 151/4550 | RT-PCR/qPCR |
| Myo-2r | GCGGTTCTTACTGTCACCCAAAG | 57 | Myocd |  | RT-PCR/qPCR |
| Pln-1 | CATGCTCTGCACTGTGACGATC | 57 | Pln | 117/4303 | RT-PCR/qPCR |
| Pln-1r | GCAGAATTGTAAGCTGGCAAGTTCC | 58 | Pln |  | RT-PCR/qPCR |
| Brm-1 | GATGAAGAGGAGTCGGAGTCAG | 57 | Smarca2 | 163/1958 | RT-PCR/qPCR |
| Brm-1r | CTTCCTGTTCCTCGTCACTGTC | 57 | Smarca2 |  | RT-PCR/qPCR |
| Brg-1 | GATCGTGGATGCTGTGATCAAGTAC | 58 | Smarca4 | 130/13654 | RT-PCR/qPCR |
| Brg-1r | GAAGTCCACAGGCTTTCGGATG | 57 | Smarca4 |  | RT-PCR/qPCR |
| SRF-2 | GAGTGCCACTGGCTTTGAAGAG | 57 | Srf | 166/1933 | RT-PCR/qPCR |
| SRF-2r | GACTTGCATGGTGGTAGAGGTG | 57 | Srf |  | RT-PCR/qPCR |
| SM22a-1 | CAGCAGTGCAGAGGACTCTAATGG | 59 | Tagln | 131/449 | RT-PCR/qPCR |
| SM22a-1r | GTTGGCTGTCTGTGAAGTCCCTC | 59 | Tagln |  | RT-PCR/qPCR |
| Tln-1 | CAACCAGCTGACCAGTGACTATG | 57 | Tln1 | 112/1229 | RT-PCR/qPCR |
| Tln-1r | CAGCTCCTGTACTCGGTGTTTG | 57 | Tln1 |  | RT-PCR/qPCR |
| Vcam-1 | CAGGATGCCGGCATATACGAGTG | 59 | Vcam1 | 131/3447 | RT-PCR/qPCR |
| Vcam-1r | GCAGTAGAGTGCAAGGAGTTCG | 57 | Vcam1 |  | RT-PCR/qPCR |
| Vnn-1 | GTCTGAGAAGCGAGCAGATGAG | 57 | Vnn1 | 142/2584 | RT-PCR/qPCR |
| Vnn-1r | GTCCACTGAACTACCACAGGTG | 58 | Vnn1 |  | RT-PCR/qPCR |
| U1A | CTGCATAATTTGTGGTAGTGGG | 53 | piRNA24102 | 130 | RT-PCR/qPCR |
| U24 | AACCACCAAGATCGCTGATGC | 54 | Snord243 | 122 | RT-PCR/qPCR |
| U43 | TTGACGGGCGGACAGAAACT | 54 | Snord43 | 145 | RT-PCR/qPCR |
| U58A | GACACCTTTGGATTTACCGTGAA | 53 | Snord58A3 | 144 | RT-PCR/qPCR |
| snoR-202 | TTGAACCCTTTTCCATCTGATG | 51 | Snord68 | 122 | RT-PCR/qPCR |
| miR-190 | TGATATGTTTGATATATTAG | 39 | miR-190 | 122 | RT-PCR/qPCR |
| let-7a*/c* | CTATACAATCTACTGTCTT | 43 | let-7a*/c* | 121 | RT-PCR/qPCR |
| miR-369-3p | AATAATACATGGTTGATCT | 43 | miR-369-3p | 119 | RT-PCR/qPCR |
| miR-126-5p | CATTATTACTTTTGGTACG | 43 | miR-126-5p | 121 | RT-PCR/qPCR |
| miR-1* | ACATACTTCTTTATGTACC | 43 | miR-1* | 121 | RT-PCR/qPCR |
| miR-26b | TTCAAGTAATTCAGGATAG | 43 | miR-26b | 121 | RT-PCR/qPCR |
| miR-142-5p | CATAAAGTAGAAAGCACTA | 44 | miR-142-5p | 121 | RT-PCR/qPCR |
| miR-374 | ATATAATACAACCTGCTAAG | 44 | miR-374 | 121 | RT-PCR/qPCR |
| miR-805 | GAATTGATCAGGACATAG | 44 | miR-805 | 121 | RT-PCR/qPCR |
| miR-202-5p | TTCCTATGCATATACTTCT | 45 | miR-202-5p | 121 | RT-PCR/qPCR |
| miR-376c | AACATAGAGGAAATTTCAC | 45 | miR-376c | 121 | RT-PCR/qPCR |
| let-7f | TGAGGTAGTAGATTGTATAG | 45 | let-7f | 120 | RT-PCR/qPCR |
| miR-469 | TTCATTGATCTTGGTGT | 45 | miR-469 | 120 | RT-PCR/qPCR |
| let-7f* | CTATACAATCTATTGCCTTC | 45 | let-7f* | 122 | RT-PCR/qPCR |
| miR-101a | TACAGTACTGTGATAACTG | 46 | miR-101a | 122 | RT-PCR/qPCR |
| miR-872 | AAGGTTACTTGTTAGTTCA | 46 | miR-872 | 122 | RT-PCR/qPCR |
| miR-467e | ATAAGTGTGAGCATGTATAT | 46 | miR-467e | 122 | RT-PCR/qPCR |
| miR-9* | ATAAAGCTAGATAACCGAAA | 46 | miR-9* | 122 | RT-PCR/qPCR |
| miR-1 | TGGAATGTAAAGAAGTATGT | 46 | miR-1 | 122 | RT-PCR/qPCR |
| miR-340-5p | TTATAAAGCAATGAGACTGA | 46 | miR-340-5p | 122 | RT-PCR/qPCR |
| miR-98 | TGAGGTAGTAAGTTGTATTG | 46 | miR-98 | 121 | RT-PCR/qPCR |
| miR-215 | ATGACCTATGATTTGACAG | 47 | miR-215 | 121 | RT-PCR/qPCR |
| miR-384-3p | ATTCCTAGAAATTGTTCACA | 47 | miR-384-3p | 121 | RT-PCR/qPCR |
| miR-135a | TATGGCTTTTTATTCCTATGT | 47 | miR-135a | 121 | RT-PCR/qPCR |
| miR-26a* | CCTGTTCTTGATTACTTGT | 47 | miR-26a* | 121 | RT-PCR/qPCR |
| miR-142-3p | TGTAGTGTTTCCTACTTTATG | 47 | miR-142-3p | 123 | RT-PCR/qPCR |
| miR-379* | TATGTAACATGGTCCACT | 47 | miR-379* | 123 | RT-PCR/qPCR |
| let-7a | TGAGGTAGTAGGTTGTATAG | 47 | let-7a | 121 | RT-PCR/qPCR |
| miR-195 | TAGCAGCACAGAAATATTG | 47 | miR-195 | 121 | RT-PCR/qPCR |
| miR-126 | TCGTACCGTGAGTAATAAT | 47 | miR-126 | 121 | RT-PCR/qPCR |
| miR-136 | ACTCCATTTGTTTTGATGAT | 48 | miR-136 | 122 | RT-PCR/qPCR |
| miR-376a* | GGTAGATTCTCCTTCTATGA | 48 | miR-376a* | 122 | RT-PCR/qPCR |
| miR-21 | TAGCTTATCAGACTGATGTT | 48 | miR-21 | 122 | RT-PCR/qPCR |
| miR-101b | TACAGTACTGTGATAGCTG | 48 | miR-101b | 122 | RT-PCR/qPCR |
| miR-200b | TAATACTGCCTGGTAATGAT | 48 | miR-200b | 122 | RT-PCR/qPCR |
| miR-410 | AATATAACACAGATGGCCT | 48 | miR-410 | 122 | RT-PCR/qPCR |
| miR-9 | TCTTTGGTTATCTAGCTGTAT | 48 | miR-9 | 122 | RT-PCR/qPCR |
| miR-335-5p | TCAAGAGCAATAACGAAAAAT | 48 | miR-335-5p | 122 | RT-PCR/qPCR |
| miR-380-3p | TATGTAGTATGGTCCACATC | 48 | miR-380-3p | 122 | RT-PCR/qPCR |
| let-7g | TGAGGTAGTAGTTTGTACAG | 48 | let-7g | 122 | RT-PCR/qPCR |
| miR-466a-5p | TATGTGTGTGTACATGTACA | 48 | miR-466a-5p | 122 | RT-PCR/qPCR |
| miR-376b | ATCATAGAGGAACATCCAC | 48 | miR-376b | 122 | RT-PCR/qPCR |
| miR-411 | TAGTAGACCGTATAGCGTA | 48 | miR-411 | 122 | RT-PCR/qPCR |
| miR-29a* | ACTGATTTCTTTTGGTGTTC | 49 | miR-29a* | 122 | RT-PCR/qPCR |
| miR-301a | CAGTGCAATAGTATTGTCAAA | 49 | miR-301a | 122 | RT-PCR/qPCR |
| miR-26a | TTCAAGTAATCCAGGATAGG | 49 | miR-26a | 122 | RT-PCR/qPCR |
| miR-376b* | GTGGATATTCCTTCTATGGT | 49 | miR-376b* | 122 | RT-PCR/qPCR |
| miR-582-5p | TACAGTTGTTCAACCAGTTA | 49 | miR-582-5p | 121 | RT-PCR/qPCR |
| miR-337-3p | TTCAGCTCCTATATGATGC | 49 | miR-337-3p | 121 | RT-PCR/qPCR |
| miR-218 | TTGTGCTTGATCTAACCAT | 49 | miR-218 | 121 | RT-PCR/qPCR |
| miR-466b/c/e-3p | TATACATACACGCACACATAA | 49 | miR-466b/c/e-3p | 121 | RT-PCR/qPCR |
| miR-30b | TGTAAACATCCTACACTCAG | 49 | miR-30b | 121 | RT-PCR/qPCR |
| miR-376a | ATCGTAGAGGAAAATCCAC | 49 | miR-376a | 123 | RT-PCR/qPCR |
| miR-145* | GGATTCCTGGAAATACTGT | 49 | miR-145* | 121 | RT-PCR/qPCR |
| miR-146b | TGAGAACTGAATTCCATAGG | 49 | miR-146b | 121 | RT-PCR/qPCR |
| miR-10a* | CAAATTCGTATCTAGGGGAA | 49 | miR-10a* | 121 | RT-PCR/qPCR |
| miR-429 | TAATACTGTCTGGTAATGCC | 49 | miR-429 | 121 | RT-PCR/qPCR |
| miR-365 | TAATGCCCCTAAAAATCCTT | 49 | miR-365 | 121 | RT-PCR/qPCR |
| miR-325 | TTTATTGAGCACCTCCTATC | 49 | miR-325 | 123 | RT-PCR/qPCR |
| miR-369-5p | AGATCGACCGTGTTATATTC | 49 | miR-369-5p | 123 | RT-PCR/qPCR |
| miR-670* | CCTCATATCCATTCAGGAG | 49 | miR-670* | 121 | RT-PCR/qPCR |
| miR-186 | CAAAGAATTCTCCTTTTGGG | 49 | miR-186 | 123 | RT-PCR/qPCR |
| miR-379 | TGGTAGACTATGGAACGTA | 49 | miR-379 | 123 | RT-PCR/qPCR |
| miR-135b | TATGGCTTTTCATTCCTATGT | 49 | miR-135b | 123 | RT-PCR/qPCR |
| miR-7a | TGGAAGACTAGTGATTTTGTT | 49 | miR-7a | 121 | RT-PCR/qPCR |
| miR-467b | GTAAGTGCCTGCATGTATA | 49 | miR-467b | 123 | RT-PCR/qPCR |
| miR-669d | ACTTGTGTGTGCATGTATAT | 49 | miR-669d | 123 | RT-PCR/qPCR |
| miR-669l | AGTTGTGTGTGCATGTATAT | 49 | miR-669l | 123 | RT-PCR/qPCR |
| miR-1839-5p | AAGGTAGATAGAACAGGTCT | 49 | miR-1839-5p | 123 | RT-PCR/qPCR |
| miR-136* | ATCATCGTCTCAAATGAGTC | 50 | miR-136* | 121 | RT-PCR/qPCR |
| miR-15b* | CGAATCATTATTTGCTGCTC | 50 | miR-15b* | 121 | RT-PCR/qPCR |
| miR-539 | GGAGAAATTATCCTTGGTGT | 50 | miR-539 | 122 | RT-PCR/qPCR |
| miR-592 | TTGTGTCAATATGCGATGAT | 50 | miR-592 | 122 | RT-PCR/qPCR |
| miR-153 | TTGCATAGTCACAAAAGTGA | 50 | miR-153 | 122 | RT-PCR/qPCR |
| miR-384-5p | TGTAAACAATTCCTAGGCAAT | 50 | miR-384-5p | 122 | RT-PCR/qPCR |
| miR-496 | TGAGTATTACATGGCCAATC | 50 | miR-496 | 122 | RT-PCR/qPCR |
| miR-1839-3p | AGACCTACTTATCTACCAACA | 50 | miR-1839-3p | 122 | RT-PCR/qPCR |
| miR-450b-5p | TTTTGCAGTATGTTCCTGAA | 50 | miR-450b-5p | 122 | RT-PCR/qPCR |
| miR-467a | TAAGTGCCTGCATGTATATG | 50 | miR-467a | 122 | RT-PCR/qPCR |
| miR-466b-5p | GATGTGTGTGTACATGTACA | 50 | miR-466b-5p | 122 | RT-PCR/qPCR |
| miR-1193 | TAGGTCACCCGTTTTACTA | 50 | miR-1193 | 122 | RT-PCR/qPCR |
| miR-451 | AAACCGTTACCATTACTGAG | 50 | miR-451 | 122 | RT-PCR/qPCR |
| miR-297b-5p | ATGTATGTGTGCATGAACAT | 50 | miR-297b-5p | 122 | RT-PCR/qPCR |
| miR-137 | TTATTGCTTAAGAATACGCGT | 50 | miR-137 | 122 | RT-PCR/qPCR |
| let-7e | TGAGGTAGGAGGTTGTATAG | 50 | let-7e | 122 | RT-PCR/qPCR |
| miR-30e* | CTTTCAGTCGGATGTTTACA | 50 | miR-30e* | 122 | RT-PCR/qPCR |
| miR-203 | GTGAAATGTTTAGGACCACT | 50 | miR-203 | 122 | RT-PCR/qPCR |
| miR-450a-5p | TTTTGCGATGTGTTCCTAAT | 50 | miR-450a-5p | 122 | RT-PCR/qPCR |
| miR-16 | TAGCAGCACGTAAATATTGG | 50 | miR-16 | 122 | RT-PCR/qPCR |
| miR-7b | TGGAAGACTTGTGATTTTGTT | 50 | miR-7b | 122 | RT-PCR/qPCR |
| miR-223 | TGTCAGTTTGTCAAATACCC | 50 | miR-223 | 122 | RT-PCR/qPCR |
| miR-669o | AGTTGTGTGTGCATGTTTAT | 50 | miR-669o | 122 | RT-PCR/qPCR |
| miR-300* | GAGAGGTTATCCTTTGTGTG | 50 | miR-300* | 122 | RT-PCR/qPCR |
| miR-411* | TATGTAACACGGTCCACTAA | 50 | miR-411* | 122 | RT-PCR/qPCR |
| miR-30c | TGTAAACATCCTACACTCTCA | 50 | miR-30c | 122 | RT-PCR/qPCR |
| miR-107* | AGCTTCTTTACAGTGTTGC | 51 | miR-107* | 122 | RT-PCR/qPCR |
| let-7c | TGAGGTAGTAGGTTGTATGG | 51 | let-7c | 122 | RT-PCR/qPCR |
| miR-219 | TGATTGTCCAAACGCAATT | 51 | miR-219 | 122 | RT-PCR/qPCR |
| let-7d | AGAGGTAGTAGGTTGCATAG | 51 | let-7d | 122 | RT-PCR/qPCR |
| miR-200a | TAACACTGTCTGGTAACGAT | 51 | miR-200a | 122 | RT-PCR/qPCR |
| miR-15a | TAGCAGCACATAATGGTTTG | 51 | miR-15a | 122 | RT-PCR/qPCR |
| miR-154* | AATCATACACGGTTGACCTA | 51 | miR-154* | 122 | RT-PCR/qPCR |
| miR-322 | CAGCAGCAATTCATGTTTTG | 51 | miR-322 | 122 | RT-PCR/qPCR |
| miR-27b | TTCACAGTGGCTAAGTTCT | 51 | miR-27b | 122 | RT-PCR/qPCR |
| miR-19a | TGTGCAAATCTATGCAAAACT | 51 | miR-19a | 122 | RT-PCR/qPCR |
| miR-224 | TAAGTCACTAGTGGTTCCG | 51 | miR-224 | 122 | RT-PCR/qPCR |
| miR-29b | TAGCACCATTTGAAATCAGTG | 51 | miR-29b | 122 | RT-PCR/qPCR |
| miR-30e | TGTAAACATCCTTGACTGGA | 51 | miR-30e | 122 | RT-PCR/qPCR |
| miR-421 | ATCAACAGACATTAATTGGGC | 51 | miR-421 | 122 | RT-PCR/qPCR |
| miR-455* | TATGTGCCTTTGGACTACAT | 51 | miR-455* | 122 | RT-PCR/qPCR |
| miR-23b | ATCACATTGCCAGGGATTA | 51 | miR-23b | 122 | RT-PCR/qPCR |
| miR-493* | TTGTACATGGTAGGCTTTCA | 51 | miR-493* | 122 | RT-PCR/qPCR |
| miR-20a | TAAAGTGCTTATAGTGCAGGT | 51 | miR-20a | 122 | RT-PCR/qPCR |
| miR-148a | TCAGTGCACTACAGAACTTT | 51 | miR-148a | 122 | RT-PCR/qPCR |
| miR-152 | TCAGTGCATGACAGAACTT | 52 | miR-152 | 122 | RT-PCR/qPCR |
| miR-669c | ATAGTTGTGTGTGGATGTGT | 52 | miR-669c | 122 | RT-PCR/qPCR |
| miR-28 | AAGGAGCTCACAGTCTATTG | 52 | miR-28 | 121 | RT-PCR/qPCR |
| miR-15b | TAGCAGCACATCATGGTTTA | 52 | miR-15b | 121 | RT-PCR/qPCR |
| miR-10a | TACCCTGTAGATCCGAATTTG | 52 | miR-10a | 123 | RT-PCR/qPCR |
| let-7i | TGAGGTAGTAGTTTGTGCTG | 52 | let-7i | 121 | RT-PCR/qPCR |
| miR-664 | TATTCATTTACTCCCCAGCC | 52 | miR-664 | 123 | RT-PCR/qPCR |
| miR-495 | AAACAAACATGGTGCACTTC | 52 | miR-495 | 121 | RT-PCR/qPCR |
| miR-143 | TGAGATGAAGCACTGTAGC | 52 | miR-143 | 121 | RT-PCR/qPCR |
| miR-708 | AAGGAGCTTACAATCTAGCTG | 52 | miR-708 | 121 | RT-PCR/qPCR |
| miR-872* | TGAACTATTGCAGTAGCCTC | 52 | miR-872* | 123 | RT-PCR/qPCR |
| miR-140 | CAGTGGTTTTACCCTATGGT | 52 | miR-140 | 121 | RT-PCR/qPCR |
| miR-148b | TCAGTGCATCACAGAACTTT | 52 | miR-148b | 123 | RT-PCR/qPCR |
| miR-362-3p | AACACACCTGTTCAAGGATT | 52 | miR-362-3p | 121 | RT-PCR/qPCR |
| miR-10b | TACCCTGTAGAACCGAATTTG | 52 | miR-10b | 123 | RT-PCR/qPCR |
| miR-23a | ATCACATTGCCAGGGATTT | 52 | miR-23a | 121 | RT-PCR/qPCR |
| miR-350 | TTCACAAAGCCCATACACTT | 52 | miR-350 | 123 | RT-PCR/qPCR |
| miR-27a | TTCACAGTGGCTAAGTTCC | 52 | miR-27a | 123 | RT-PCR/qPCR |
| miR-338-3p | TCCAGCATCAGTGATTTTGT | 52 | miR-338-3p | 121 | RT-PCR/qPCR |
| miR-29c | TAGCACCATTTGAAATCGGT | 52 | miR-29c | 122 | RT-PCR/qPCR |
| miR-146a | TGAGAACTGAATTCCATGGG | 52 | miR-146a | 122 | RT-PCR/qPCR |
| miR-382* | TCATTCACGGACAACACTTT | 52 | miR-382* | 122 | RT-PCR/qPCR |
| miR-338-5p | AACAATATCCTGGTGCTGAG | 52 | miR-338-5p | 122 | RT-PCR/qPCR |
| miR-204 | TTCCCTTTGTCATCCTATGC | 52 | miR-204 | 123 | RT-PCR/qPCR |
| miR-503* | GAGTATTGTTTCCACTGCCT | 52 | miR-503* | 122 | RT-PCR/qPCR |
| miR-361 | TTATCAGAATCTCCAGGGGT | 52 | miR-361 | 122 | RT-PCR/qPCR |
| miR-130a | CAGTGCAATGTTAAAAGGGC | 53 | miR-130a | 123 | RT-PCR/qPCR |
| miR-329* | AGAGGTTTTCTGGGTCTCT | 53 | miR-329* | 122 | RT-PCR/qPCR |
| miR-106b | TAAAGTGCTGACAGTGCAG | 53 | miR-106b | 122 | RT-PCR/qPCR |
| miR-101b* | TCGGTTATCATGGTACCGA | 53 | miR-101b* | 122 | RT-PCR/qPCR |
| miR-449a | TGGCAGTGTATTGTTAGCTG | 53 | miR-449a | 123 | RT-PCR/qPCR |
| miR-1983 | CTCACCTGGAGCATGTTTT | 53 | miR-1983 | 120 | RT-PCR/qPCR |
| miR-378 | ACTGGACTTGGAGTCAGAA | 53 | miR-378 | 122 | RT-PCR/qPCR |
| miR-669a | AGTTGTGTGTGCATGTTCAT | 53 | miR-669a | 122 | RT-PCR/qPCR |
| miR-194 | TGTAACAGCAACTCCATGTG | 53 | miR-194 | 122 | RT-PCR/qPCR |
| miR-494 | TGAAACATACACGGGAAACC | 53 | miR-494 | 122 | RT-PCR/qPCR |
| miR-381 | TATACAAGGGCAAGCTCTCT | 53 | miR-381 | 122 | RT-PCR/qPCR |
| miR-151-3p | CTAGACTGAGGCTCCTTGA | 53 | miR-151-3p | 122 | RT-PCR/qPCR |
| miR-873 | GCAGGAACTTGTGAGTCTC | 53 | miR-873 | 122 | RT-PCR/qPCR |
| mir-325* | CCTAGTAGGTGCTCAGTAAGT | 53 | mir-325* | 122 | RT-PCR/qPCR |
| miR-377 | TGAATCACACAAAGGCAACTT | 53 | miR-377 | 120 | RT-PCR/qPCR |
| miR-30a | TGTAAACATCCTCGACTGGA | 53 | miR-30a | 123 | RT-PCR/qPCR |
| miR-467d | TAAGTGCGCGCATGTATATG | 53 | miR-467d | 122 | RT-PCR/qPCR |
| miR-132 | TAACAGTCTACAGCCATGGT | 53 | miR-132 | 123 | RT-PCR/qPCR |
| miR-382 | GAAGTTGTTCGTGGTGGATT | 53 | miR-382 | 123 | RT-PCR/qPCR |
| miR-433* | TACGGTGAGCCTGTCATTAT | 53 | miR-433* | 122 | RT-PCR/qPCR |
| miR-28* | CACTAGATTGTGAGCTGCTG | 53 | miR-28* | 122 | RT-PCR/qPCR |
| miR-26b* | CCTGTTCTCCATTACTTGGC | 53 | miR-26b* | 122 | RT-PCR/qPCR |
| miR-18a | TAAGGTGCATCTAGTGCAGAT | 53 | miR-18a | 122 | RT-PCR/qPCR |
| miR-30a* | CTTTCAGTCGGATGTTTGCA | 53 | miR-30a* | 122 | RT-PCR/qPCR |
| miR-140* | TACCACAGGGTAGAACCAC | 54 | miR-140* | 122 | RT-PCR/qPCR |
| let-7e* | CTATACGGCCTCCTAGCTTT | 54 | let-7e* | 122 | RT-PCR/qPCR |
| miR-34c | AGGCAGTGTAGTTAGCTGATT | 54 | miR-34c | 122 | RT-PCR/qPCR |
| let-7b | TGAGGTAGTAGGTTGTGTGG | 54 | let-7b | 122 | RT-PCR/qPCR |
| miR-34b-3p | AATCACTAACTCCACTGCCA | 54 | miR-34b-3p | 122 | RT-PCR/qPCR |
| miR-222 | AGCTACATCTGGCTACTGG | 54 | miR-222 | 122 | RT-PCR/qPCR |
| miR-551b | GCGACCCATACTTGGTTTC | 54 | miR-551b | 123 | RT-PCR/qPCR |
| miR-154 | TAGGTTATCCGTGTTGCCTT | 54 | miR-154 | 122 | RT-PCR/qPCR |
| miR-669b/m-5p | AGTTTTGTGTGCATGTGCAT | 54 | miR-669b/m-5p | 122 | RT-PCR/qPCR |
| miR-29a | TAGCACCATCTGAAATCGGT | 54 | miR-29a | 122 | RT-PCR/qPCR |
| miR-181d | AACATTCATTGTTGTCGGTGG | 54 | miR-181d | 122 | RT-PCR/qPCR |
| miR-199b/a-3p | ACAGTAGTCTGCACATTGGT | 54 | miR-199b/a-3p | 122 | RT-PCR/qPCR |
| miR-24-1* | GTGCCTACTGAGCTGATATCA | 54 | miR-24-1* | 122 | RT-PCR/qPCR |
| miR-19b | TGTGCAAATCCATGCAAAACT | 54 | miR-19b | 121 | RT-PCR/qPCR |
| miR-383 | AGATCAGAAGGTGACTGTGG | 54 | miR-383 | 121 | RT-PCR/qPCR |
| miR-434-5p | GCTCGACTCATGGTTTGAAC | 54 | miR-434-5p | 121 | RT-PCR/qPCR |
| miR-99a | AACCCGTAGATCCGATCTTG | 54 | miR-99a | 121 | RT-PCR/qPCR |
| miR-505* | GGGAGCCAGGAAGTATTGAT | 54 | miR-505* | 121 | RT-PCR/qPCR |
| miR-466d-5p | TGTGTGTGCGTACATGTACA | 54 | miR-466d-5p | 121 | RT-PCR/qPCR |
| miR-487b | AATCGTACAGGGTCATCCAC | 54 | miR-487b | 121 | RT-PCR/qPCR |
| miR-181c | AACATTCAACCTGTCGGTGA | 54 | miR-181c | 121 | RT-PCR/qPCR |
| miR-342-5p | AGGGGTGCTATCTGTGATTG | 54 | miR-342-5p | 121 | RT-PCR/qPCR |
| miR-100 | AACCCGTAGATCCGAACTTG | 54 | miR-100 | 121 | RT-PCR/qPCR |
| miR-329 | AACACACCCAGCTAACCTTT | 54 | miR-329 | 122 | RT-PCR/qPCR |
| miR-151-5p | TCGAGGAGCTCACAGTCTA | 54 | miR-151-5p | 123 | RT-PCR/qPCR |
| miR-299* | TGGTTTACCGTCCCACATAC | 54 | miR-299* | 123 | RT-PCR/qPCR |
| let-7d* | CTATACGACCTGCTGCCTTT | 55 | let-7d* | 122 | RT-PCR/qPCR |
| miR-323-3p | CACATTACACGGTCGACCT | 55 | miR-323-3p | 122 | RT-PCR/qPCR |
| miR-540-3p | AGGTCAGAGGTCGATCCT | 55 | miR-540-3p | 122 | RT-PCR/qPCR |
| miR-1981 | CATCTAACCCTGGCCTTTGA | 55 | miR-1981 | 122 | RT-PCR/qPCR |
| miR-322* | AAACATGAAGCGCTGCAAC | 55 | miR-322* | 122 | RT-PCR/qPCR |
| miR-22 | AAGCTGCCAGTTGAAGAACT | 55 | miR-22 | 122 | RT-PCR/qPCR |
| miR-22* | AGTTCTTCAGTGGCAAGCTT | 55 | miR-22* | 123 | RT-PCR/qPCR |
| miR-125b* | ACAAGTCAGGTTCTTGGGAC | 55 | miR-125b* | 122 | RT-PCR/qPCR |
| miR-466c-5p | TGATGTGTGTGTGCATGTACA | 55 | miR-466c-5p | 122 | RT-PCR/qPCR |
| miR-485* | GTCATACACGGCTCTCCTC | 55 | miR-485* | 123 | RT-PCR/qPCR |
| miR-99a* | CAAGCTCGCTTCTATGGGT | 55 | miR-99a* | 123 | RT-PCR/qPCR |
| miR-490-5p | CCATGGATCTCCAGGTGG | 55 | miR-490-5p | 122 | RT-PCR/qPCR |
| miR-125b-5p | TCCCTGAGACCCTAACTTGT | 55 | miR-125b-5p | 122 | RT-PCR/qPCR |
| miR-25 | CATTGCACTTGTCTCGGTCT | 55 | miR-25 | 123 | RT-PCR/qPCR |
| miR-218-1* | AAACATGGTTCCGTCAAGCA | 55 | miR-218-1* | 123 | RT-PCR/qPCR |
| miR-674* | CACAGCTCCCATCTCAGAAC | 55 | miR-674* | 122 | RT-PCR/qPCR |
| miR-532-5p | CATGCCTTGAGTGTAGGACC | 55 | miR-532-5p | 122 | RT-PCR/qPCR |
| miR-676 | CCGTCCTGAGGTTGTTGAG | 55 | miR-676 | 122 | RT-PCR/qPCR |
| miR-103* | AGCTTCTTTACAGTGCTGCC | 56 | miR-103* | 123 | RT-PCR/qPCR |
| miR-128 | TCACAGTGAACCGGTCTCT | 56 | miR-128 | 122 | RT-PCR/qPCR |
| miR-362-5p | AATCCTTGGAACCTAGGTGTGA | 56 | miR-362-5p | 122 | RT-PCR/qPCR |
| miR-145 | GTCCAGTTTTCCCAGGAATCC | 56 | miR-145 | 122 | RT-PCR/qPCR |
| miR-455 | GCAGTCCACGGGCATATAC | 56 | miR-455 | 122 | RT-PCR/qPCR |
| miR-598 | TACGTCATCGTCGTCATCGT | 56 | miR-598 | 122 | RT-PCR/qPCR |
| miR-17 | CAAAGTGCTTACAGTGCAGGT | 56 | miR-17 | 122 | RT-PCR/qPCR |
| miR-124 | TAAGGCACGCGGTGAATG | 56 | miR-124 | 123 | RT-PCR/qPCR |
| miR-30d | TGTAAACATCCCCGACTGGA | 56 | miR-30d | 122 | RT-PCR/qPCR |
| miR-185 | TGGAGAGAAAGGCAGTTCCT | 56 | miR-185 | 122 | RT-PCR/qPCR |
| miR-29b-2* | CTGGTTTCACATGGTGGCTT | 56 | miR-29b-2* | 122 | RT-PCR/qPCR |
| miR-129-3p | AAGCCCTTACCCCAAAAAGC | 56 | miR-129-3p | 122 | RT-PCR/qPCR |
| miR-330 | TCTCTGGGCCTGTGTCTTAG | 56 | miR-330 | 122 | RT-PCR/qPCR |
| miR-181b | AACATTCATTGCTGTCGGTGG | 56 | miR-181b | 123 | RT-PCR/qPCR |
| miR-501-5p | AATCCTTTGTCCCTGGGTGA | 56 | miR-501-5p | 122 | RT-PCR/qPCR |
| miR-199a-5p | CCCAGTGTTCAGACTACCTGT | 56 | miR-199a-5p | 122 | RT-PCR/qPCR |
| miR-193 | AACTGGCCTACAAAGTCCCA | 56 | miR-193 | 122 | RT-PCR/qPCR |
| miR-129-5p | CTTTTTGCGGTCTGGGCTT | 56 | miR-129-5p | 122 | RT-PCR/qPCR |
| miR-150 | TCTCCCAACCCTTGTACCAG | 56 | miR-150 | 122 | RT-PCR/qPCR |
| miR-541 | AAGGGATTCTGATGTTGGTCACA | 56 | miR-541 | 125 | RT-PCR/qPCR |
| miR-300 | TATGCAAGGGCAAGCTCTCT | 56 | miR-300 | 124 | RT-PCR/qPCR |
| miR-214* | TGCCTGTCTACACTTGCTGT | 56 | miR-214* | 121 | RT-PCR/qPCR |
| miR-574-5p | TGAGTGTGTGTGTGTGAGTGT | 56 | miR-574-5p | 121 | RT-PCR/qPCR |
| miR-425 | AATGACACGATCACTCCCGTT | 56 | miR-425 | 120 | RT-PCR/qPCR |
| miR-103/107 | AGCAGCATTGTACAGGGCTAT | 56 | miR-103/107 | 121 | RT-PCR/qPCR |
| miR-188-3p | CTCCCACATGCAGGGTTTG | 56 | miR-188-3p | 121 | RT-PCR/qPCR |
| miR-34a | TGGCAGTGTCTTAGCTGGTT | 56 | miR-34a | 121 | RT-PCR/qPCR |
| miR-370* | CAGGTCACGTCTCTGCAGTTA | 57 | miR-370* | 121 | RT-PCR/qPCR |
| miR-125a-3p | ACAGGTGAGGTTCTTGGGAG | 57 | miR-125a-3p | 121 | RT-PCR/qPCR |
| miR-409-3p | GAATGTTGCTCGGTGAACCC | 57 | miR-409-3p | 121 | RT-PCR/qPCR |
| miR-205 | TCCTTCATTCCACCGGAGTC | 57 | miR-205 | 121 | RT-PCR/qPCR |
| miR-221 | AGCTACATTGTCTGCTGGGTT | 57 | miR-221 | 122 | RT-PCR/qPCR |
| miR-1198 | TATGTGTTCCTGGCTGGCTT | 57 | miR-1198 | 119 | RT-PCR/qPCR |
| miR-543 | AAACATTCGCGGTGCACTTC | 57 | miR-543 | 122 | RT-PCR/qPCR |
| miR-669f-5p | AGTTGTGTGTGCATGTGCATG | 57 | miR-669f-5p | 122 | RT-PCR/qPCR |
| miR-134 | TGTGACTGGTTGACCAGAGG | 57 | miR-134 | 122 | RT-PCR/qPCR |
| miR-361* | CCCCCAGGTGTGATTCTGAT | 57 | miR-361* | 123 | RT-PCR/qPCR |
| miR-700* | TAAGGCTCCTTCCTGTGCTTG | 57 | miR-700* | 123 | RT-PCR/qPCR |
| miR-107 | AGCAGCATTGTACAGGGCTATC | 57 | miR-107 | 122 | RT-PCR/qPCR |
| miR-497 | CAGCAGCACACTGTGGTTTG | 57 | miR-497 | 122 | RT-PCR/qPCR |
| miR-181a | AACATTCAACGCTGTCGGTGA | 57 | miR-181a | 123 | RT-PCR/qPCR |
| miR-191 | CAACGGAATCCCAAAAGCAGC | 57 | miR-191 | 123 | RT-PCR/qPCR |
| miR-425* | ATCGGGAATGTCGTGTCCG | 57 | miR-425* | 122 | RT-PCR/qPCR |
| miR-127* | CTGAAGCTCAGAGGGCTCTG | 57 | miR-127* | 122 | RT-PCR/qPCR |
| miR-103 | AGCAGCATTGTACAGGGCTATG | 57 | miR-103 | 123 | RT-PCR/qPCR |
| miR-342-3p | TCTCACACAGAAATCGCACCC | 57 | miR-342-3p | 122 | RT-PCR/qPCR |
| miR-125a-5p | TCCCTGAGACCCTTTAACCTGT | 58 | miR-125a-5p | 123 | RT-PCR/qPCR |
| miR-24 | TGGCTCAGTTCAGCAGGAAC | 58 | miR-24 | 122 | RT-PCR/qPCR |
| miR-99b | CACCCGTAGAACCGACCTTG | 58 | miR-99b | 122 | RT-PCR/qPCR |
| miR-133a/b | TTTGGTCCCCTTCAACCAGC | 58 | miR-133a/b | 123 | RT-PCR/qPCR |
| miR-409-5p | AGGTTACCCGAGCAACTTTGC | 58 | miR-409-5p | 123 | RT-PCR/qPCR |
| miR-320 | AAAAGCTGGGTTGAGAGGGC | 58 | miR-320 | 122 | RT-PCR/qPCR |
| miR-490 | CAACCTGGAGGACTCCATGC | 58 | miR-490 | 123 | RT-PCR/qPCR |
| miR-143* | GGTGCAGTGCTGCATCTCT | 58 | miR-143* | 122 | RT-PCR/qPCR |
| miR-138 | AGCTGGTGTTGTGAATCAGGC | 58 | miR-138 | 122 | RT-PCR/qPCR |
| miR-92a | TATTGCACTTGTCCCGGCC | 58 | miR-92a | 122 | RT-PCR/qPCR |
| miR-652 | AATGGCGCCACTAGGGTTG | 58 | miR-652 | 122 | RT-PCR/qPCR |
| miR-378* | CTCCTGACTCCAGGTCCTGT | 58 | miR-378* | 122 | RT-PCR/qPCR |
| miR-431 | TGTCTTGCAGGCCGTCATG | 58 | miR-431 | 120 | RT-PCR/qPCR |
| miR-99b* | CAAGCTCGTGTCTGTGGGTC | 58 | miR-99b* | 123 | RT-PCR/qPCR |
| miR-540-5p | CAAGGGTCACCCTCTGACTCT | 58 | miR-540-5p | 123 | RT-PCR/qPCR |
| miR-433 | ATCATGATGGGCTCCTCGGT | 58 | miR-433 | 121 | RT-PCR/qPCR |
| miR-133b | TTTGGTCCCCTTCAACCAGCTA | 59 | miR-133b | 124 | RT-PCR/qPCR |
| miR-93 | CAAAGTGCTGTTCGTGCAGGT | 59 | miR-93 | 122 | RT-PCR/qPCR |
| miR-149 | TCTGGCTCCGTGTCTTCACTC | 59 | miR-149 | 122 | RT-PCR/qPCR |
| miR-337-5p | GAACGGCGTCATGCAGGAG | 59 | miR-337-5p | 122 | RT-PCR/qPCR |
| miR-125b-3p | ACGGGTTAGGCTCTTGGGAG | 59 | miR-125b-3p | 122 | RT-PCR/qPCR |
| miR-127 | TCGGATCCGTCTGAGCTTGG | 59 | miR-127 | 122 | RT-PCR/qPCR |
| miR-665 | ACCAGGAGGCTGAGGTCC | 59 | miR-665 | 122 | RT-PCR/qPCR |
| let-7i* | CTGCGCAAGCTACTGCCTTG | 59 | let-7i* | 123 | RT-PCR/qPCR |
| miR-543* | AGTTGCCCGCGTGTTTTTCG | 59 | miR-543* | 122 | RT-PCR/qPCR |
| miR-331-3p | GCCCCTGGGCCTATCCTAG | 60 | miR-331-3p | 122 | RT-PCR/qPCR |
| miR-133a | TTTGGTCCCCTTCAACCAGCTG | 60 | miR-133a | 122 | RT-PCR/qPCR |
| miR-345-5p | GCTGACCCCTAGTCCAGTGC | 60 | miR-345-5p | 122 | RT-PCR/qPCR |
| miR-15a* | CAGGCCATACTGTGCTGCCT | 60 | miR-15a* | 122 | RT-PCR/qPCR |
| miR-106b* | CCGCACTGTGGGTACTTGCT | 60 | miR-106b* | 122 | RT-PCR/qPCR |
| miR-500 | AATGCACCTGGGCAAGGGTT | 60 | miR-500 | 122 | RT-PCR/qPCR |
| miR-501-3p | AATGCACCCGGGCAAGGATT | 60 | miR-501-3p | 122 | RT-PCR/qPCR |
| miR-1937b | AATCCCGGACGAGCCCC | 60 | miR-1937b | 122 | RT-PCR/qPCR |
| miR-193b | AACTGGCCCACAAAGTCCCG | 61 | miR-193b | 123 | RT-PCR/qPCR |
| miR-574-3p | CACGCTCATGCACACACCCA | 61 | miR-574-3p | 120 | RT-PCR/qPCR |
| miR-532-3p | CCTCCCACACCCAAGGCTTG | 61 | miR-532-3p | 122 | RT-PCR/qPCR |
| miR-92b | TATTGCACTCGTCCCGGCCT | 61 | miR-92b | 122 | RT-PCR/qPCR |
| miR-486 | TCCTGTACTGAGCTGCCCCG | 62 | miR-486 | 122 | RT-PCR/qPCR |
| miR-341 | TCGGTCGATCGGTCGGTCG | 62 | miR-341 | 121 | RT-PCR/qPCR |
| miR-326 | CCTCTGGGCCCTTCCTCCA | 62 | miR-326 | 123 | RT-PCR/qPCR |
| miR-324-3p | CCACTGCCCCAGGTGCTG | 62 | miR-324-3p | 122 | RT-PCR/qPCR |
| miR-339-5p | TCCCTGTCCTCCAGGAGCTCA | 62 | miR-339-5p | 121 | RT-PCR/qPCR |
| miR-744 | TGCGGGGCTAGGGCTAACAG | 62 | miR-744 | 122 | RT-PCR/qPCR |
| miR-214 | ACAGCAGGCACAGACAGGCA | 62 | miR-214 | 123 | RT-PCR/qPCR |
| miR-324-5p | CGCATCCCCTAGGGCATTGGT | 62 | miR-324-5p | 122 | RT-PCR/qPCR |
| miR-423-5p | TGAGGGGCAGAGAGCGAGACT | 62 | miR-423-5p | 121 | RT-PCR/qPCR |
| miR-328 | CTGGCCCTCTCTGCCCTTCC | 63 | miR-328 | 123 | RT-PCR/qPCR |
| miR-210 | CTGTGCGTGTGACAGCGGCT | 63 | miR-210 | 122 | RT-PCR/qPCR |
| miR-615-3p | TCCGAGCCTGGGTCTCCCTC | 63 | miR-615-3p | 124 | RT-PCR/qPCR |
| miR-351 | TCCCTGAGGAGCCCTTTGAGCC | 63 | miR-351 | 123 | RT-PCR/qPCR |
| miR-484 | TCAGGCTCAGTCCCCTCCCG | 64 | miR-484 | 122 | RT-PCR/qPCR |
| miR-431* | TGCAGGTCGTCTTGCAGGGCTT | 64 | miR-431* | 122 | RT-PCR/qPCR |
| miR-666-3p | GGCTGCAGCGTGATCGCCTG | 64 | miR-666-3p | 122 | RT-PCR/qPCR |
| miR-770-5p | AGCACCACGTGTCTGGGCCA | 64 | miR-770-5p | 124 | RT-PCR/qPCR |
| miR-668 | TGTCACTCGGCTCGGCCCACTA | 65 | miR-668 | 122 | RT-PCR/qPCR |
| miR-423-3p | AGCTCGGTCTGAGGCCCCTCA | 65 | miR-423-3p | 124 | RT-PCR/qPCR |
